# Guanosine inhibits hepatitis C virus replication and increases indel frequencies, associated with altered intracellular nucleotide pools

**DOI:** 10.1371/journal.ppat.1010210

**Published:** 2022-01-27

**Authors:** Rosario Sabariegos, Ana María Ortega-Prieto, Luis Díaz-Martínez, Ana Grande-Pérez, Carlos García Crespo, Isabel Gallego, Ana I. de Ávila, Laura Albentosa-González, María Eugenia Soria, Pablo Gastaminza, Esteban Domingo, Celia Perales, Antonio Mas

**Affiliations:** 1 Laboratorio de Virología Molecular, Centro Regional de Investigaciones Biomédicas (CRIB), Universidad de Castilla-La Mancha, Albacete, Spain; 2 Facultad de Medicina, Universidad de Castilla-La Mancha, Albacete, Spain; 3 Unidad de Biomedicina UCLM-CSIC, Albacete, Spain; 4 Centro de Biología Molecular “Severo Ochoa”, Consejo Superior de Investigaciones Científicas (CSIC)-Universidad Autónoma de Madrid (UAM), Campus de Cantoblanco, Madrid, Spain; 5 Instituto de Hortofruticultura Subtropical y Mediterránea "La Mayora," Universidad de Málaga-Consejo Superior de Investigaciones Científicas (IHMS-UMA-CSIC), Málaga, Spain; 6 Área de Genética, Facultad de Ciencias, Universidad de Málaga, Málaga, Spain; 7 Centro de Investigación Biomédica en Red de Enfermedades Hepáticas y Digestivas (CIBERehd) del Instituto de Salud Carlos III, Madrid, Spain; 8 Department of Clinical Microbiology, IIS-Fundación Jiménez Díaz, UAM, Madrid, Spain; 9 Department of Cellular and Molecular Biology, Centro Nacional de Biotecnología, Consejo Superior de Investigaciones Científicas (CSIC), Campus de Cantoblanco, Madrid, Spain; 10 Facultad de Farmacia, Universidad de Castilla-La Mancha, Albacete, Spain; Institut Pasteur, FRANCE

## Abstract

In the course of experiments aimed at deciphering the inhibition mechanism of mycophenolic acid and ribavirin in hepatitis C virus (HCV) infection, we observed an inhibitory effect of the nucleoside guanosine (Gua). Here, we report that Gua, and not the other standard nucleosides, inhibits HCV replication in human hepatoma cells. Gua did not directly inhibit the *in vitro* polymerase activity of NS5B, but it modified the intracellular levels of nucleoside di- and tri-phosphates (NDPs and NTPs), leading to deficient HCV RNA replication and reduction of infectious progeny virus production. Changes in the concentrations of NTPs or NDPs modified NS5B RNA polymerase activity *in vitro*, in particular *de novo* RNA synthesis and template switching. Furthermore, the Gua-mediated changes were associated with a significant increase in the number of indels in viral RNA, which may account for the reduction of the specific infectivity of the viral progeny, suggesting the presence of defective genomes. Thus, a proper NTP:NDP balance appears to be critical to ensure HCV polymerase fidelity and minimal production of defective genomes.

## Author summary

Ribonucleoside metabolism is essential for replication of RNA viruses. In this article we describe the antiviral activity of the natural ribonucleoside guanosine (Gua). We demonstrate that hepatitis C virus (HCV) replication is inhibited in the presence of increasing concentrations of this ribonucleoside and that this inhibition does not occur as a consequence of a direct inhibition of HCV polymerase. Cells exposed to increasing concentrations of Gua show imbalances in the intracellular concentrations of nucleoside-diphosphates and triphosphates and as the virus is passaged in these cells, it accumulates mutations that reduce its infectivity and decimate its normal spreading capacity.

## Introduction

Positive-sense single-stranded RNA viruses [(+)ssRNA viruses] are the most abundant pathogens for humans. The hepatitis C virus (HCV) is a hepacivirus that belongs to the *Flaviviridae* family of (+)ssRNA viruses. The HCV genome encodes information for the synthesis of ten proteins: core (C), envelope glycoproteins (E1 and E2), an ion channel (p7), NS2 protease, protease/helicase NS3 (and its cofactor NS4A), membrane-associated protein NS4B, regulator of viral replication NS5A, and RNA-dependent RNA-polymerase NS5B [1].

There are several ways to approach the control of RNA viral diseases. Inhibition of HCV functions by direct-acting antiviral agents (DAAs) has yielded sustained virological responses of about 98% [2,3]. Thus, HCV infection may be targeted for eradication by the combined use of different DAAs directed to viral proteins. However, access to this treatment is not affordable in countries with high prevalence rates, and an effective prophylactic vaccine is not available, making global HCV eradication difficult. Consequently, treatment with a combination of pegylated interferon-alpha (PEG-IFNα) plus ribavirin (Rib) is still in use in several countries with high prevalence rates of HCV infection [4].

Rib displays several mechanisms of antiviral activity [5], a major one being the inhibition of inosine-5’-monophosphate (IMP) dehydrogenase (IMPDH), which converts IMP to xanthosine monophosphate (XMP) and thus is involved in the *de novo* biosynthesis of GTP [6]. Rib also exerts its antiviral activity through lethal mutagenesis [7–10]. In the course of our experiments on the effect of mycophenolic acid and Rib on HCV clonal population HCV p0 [11] we observed that the presence of guanosine (Gua) during viral replication produced a decrease of up to 100 times in infectious progeny production. Although there are Gua derivatives that have antiviral properties, including Rib itself, natural Gua has never been identified as having antiviral activity [5]. The objective of the present study was to quantify the inhibitory role of Gua on HCV, its specificity, and its mechanism of action. We show that i) Gua inhibits infectious HCV progeny production but does not inhibit directly the HCV polymerase; ii) Gua alters the intracellular pools of di- and triphosphate ribonucleosides (NDP and NTP); iii) the imbalance of the concentrations of NDP and NTP results in the inhibition of HCV polymerase activity *in vitro*, and iv) Gua treatment is associated with an increase of indel frequency in progeny HCV RNA. The results provide evidence of a metabolism-dependent mechanism of generation of defective HCV genomes.

## Results

### Effect of ribonucleosides on HCV replication

Before studying the possible anti-HCV effect of natural nucleosides we determined their cytotoxicity (CC_50_) on Huh-7.5 reporter cells. The cytotoxicity of Gua, adenosine (Ade), cytidine (Cyt) or uridine (Uri) was analyzed in semiconfluent cell monolayers by exposing cells to different nucleoside concentrations (from 0 μM to 800 μM). Cell viability (CC_50_) was monitored after 72 h of treatment (Table 1) as described in Materials and Methods. Only Ade showed a modest cytotoxicity in the range of concentrations tested.

**Table 1:** Effects of nucleosides on cell viability and HCV replication. CC_50_, IC_50_, and therapeutic index (TI, CC_50_/IC_50_) values are shown for Adenosine (Ade), Cytidine (Cyt), Guanosine (Gua), and Uridine (Uri) in Huh-7.5 reporter cells.

	CC_50_ (μM)	IC_50_ (μM)	TI
Ade	641 ± 40	108 ± 7	5.9
Cyt	> 800	> 800	n.d.
Gua	> 800	164 ± 2.4	≥ 4.9
Uri	> 800	> 800	n.d.

To quantify the inhibition of HCV infectious progeny production in the presence of nucleosides (IC_50_), Huh-7.5 reporter cells were infected with HCV p0 at a multiplicity of infection (m.o.i.) of 0.05–0.1 TCID_50_ per cell in the presence of increasing concentrations of the corresponding nucleoside, and infectious progeny production was measured as described in Materials and Methods. A decrease in the production of HCV infectious progeny was observed for Gua and Ade, whereas Cyt and Uri did not show any effect (Table 1). These data yield a therapeutic index (TI), defined as CC_50_/IC_50_, of 5.9 and ≥ 4.9 for Ade and Gua, respectively (Table 1).

**Fig 1:**
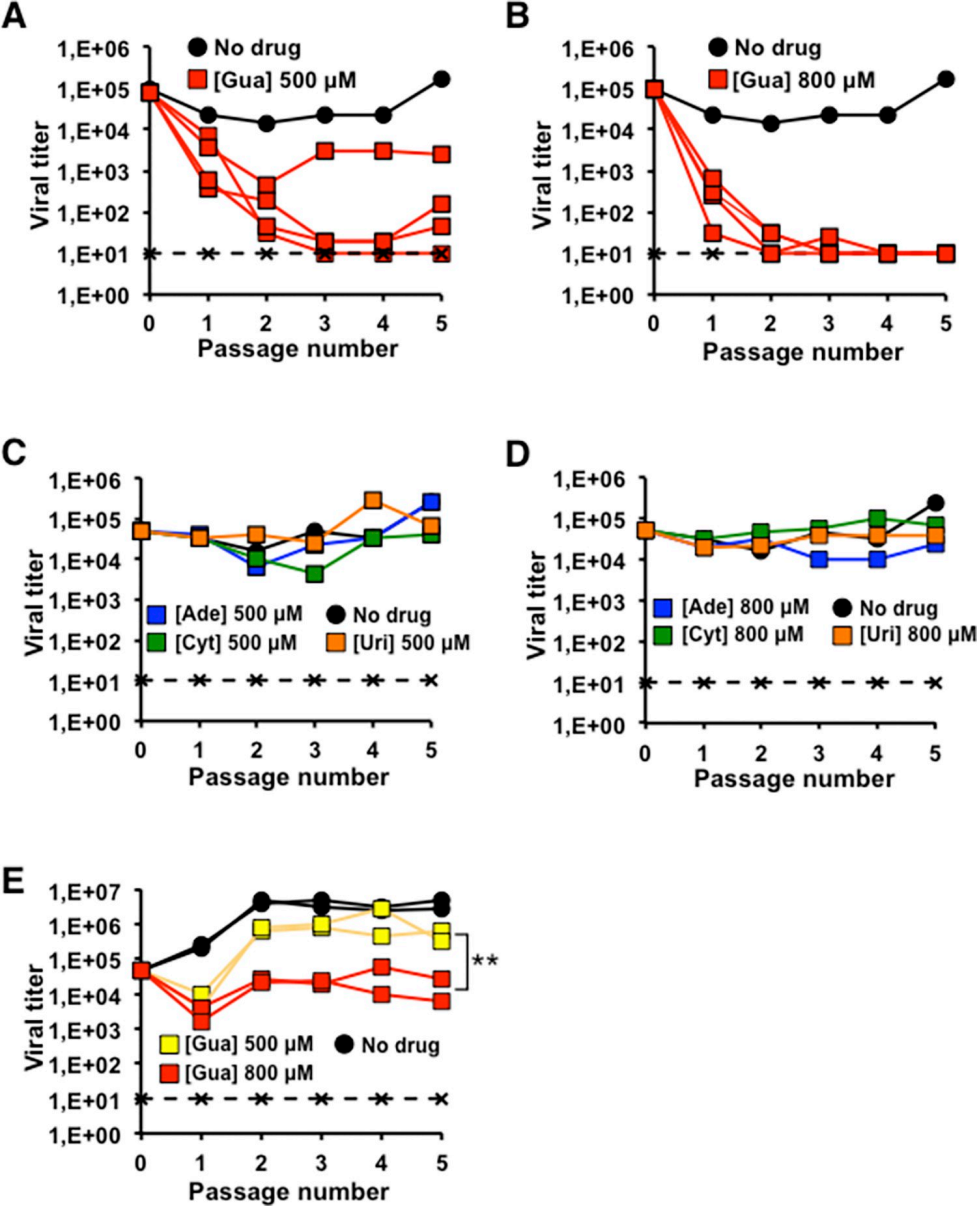
Effect of ribonucleosides on HCV replication. **(A)** and **(B)** Effect of guanosine (Gua) on HCV p0 replication. Infectious progeny obtained in the presence of Gua 500 μM (A) and Gua 800 μM (B); four replicas for each condition are shown (red squares). HCV p0 titer in the absence of treatment (black circles) and values for a HCV lethal mutant GNN (black crosses) are also shown (see Methods). Each passage was done using a fixed volume. **(C)** and **(D)** Effect of adenosine (Ade, blue squares), cytidine (Cyt, green squares), and uridine (Uri, orange squares) on HCV p0 replication. Infectious progeny obtained in the presence of the corresponding nucleoside at 500 μM (C) and at 800 μM (D). HCV p0 viral titer in the absence of treatment (black circles) and values for a HCV lethal mutant GNN (black crosses) are also shown. **(E)** HCV p100 viral titer in the absence (black circles) or presence of Gua 500 μM (yellow symbols), and Gua 800 μM (red symbols). Significance (two-way ANOVA test): **p<0.005. Two replicates each one performed in triplicate are shown for each condition in presence of nucleosides. The discontinuous horizontal line marks the limit of detection of virus infectivity. Procedures for serial infections and titration of infectivity are detailed in Materials and Methods.

To further explore the effect of ribonucleosides on HCV replication, HCV p0 was subjected to 5 serial passages in Huh-7.5 reporter cells, using an initial m.o.i. of 0.05 TCID_50_ per cell, both in the absence and in the presence of ribonucleosides at 500 μM and 800 μM (Fig 1). Results show a consistent decrease in progeny infectivity as a result of Gua treatment (Fig 1A and 1B), but a sustained viral replication in the presence of Ade, Cyt or Uri (Fig 1C and 1D). In the presence of 500 μM Gua, a decrease in infectivity was detected although only one of the four replicates yielded values below the detection limit (Fig 1A). A sustained drop in HCV infectivity by Gua 800 μM was achieved, which was not detected between passages 2 and 4 in all replicates (Fig 1B). Therefore, Gua was the only nucleoside that showed antiviral activity without cytotoxicity.

**Fig 2:**
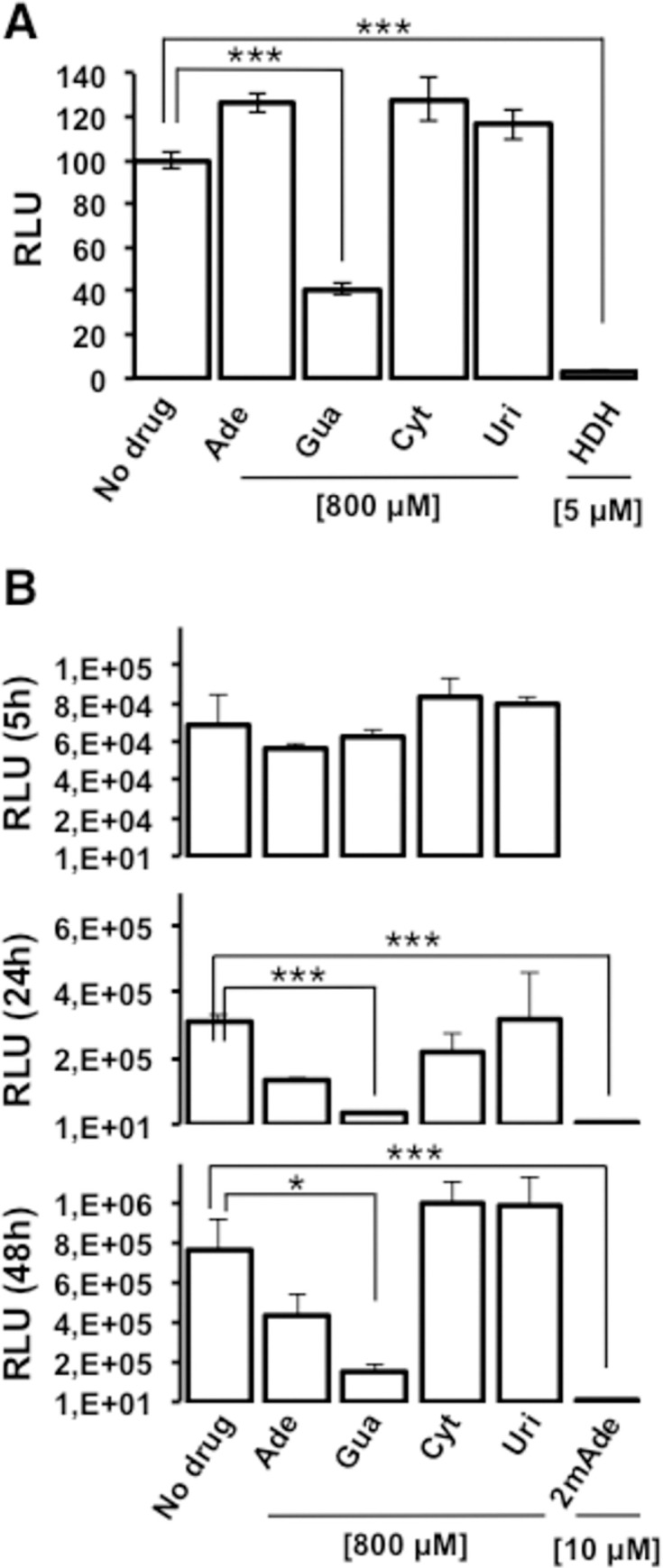
Treatment with Gua causes a reduction in the efficiency of early aspects of the infection. **(A)** Impact of nucleoside treatment on single cycle trans-complemented HCV particles (HCVtcp) infection efficiency. Huh-7 cells were pre-treated with 800 μM of nucleoside for 20 hours before inoculation with HCVtcp in the presence or absence of the nucleosides. As a positive inhibition control, target cells were treated with the entry inhibitor hydroxyzine (HDH) at the time of infection (5μM). Single cycle infection efficiency was determined by measuring luciferase activity in total cell extracts 48 hours post-inoculation. **(B)** Huh-7 cells were pre-treated with 800 μM of nucleoside for 20 hours before transfection with in vitro-transcribed subgenomic viral RNA-containing liposomes in the presence or absence of the nucleosides. As a positive inhibition control, target cells were treated with the replication inhibitor 2´-c-methyladenosine (2mAde) (10 μM). Primary translation (5 hours) and RNA replication efficiency (24 and 48 hours) was determined by measuring luciferase activity in total cell extracts at different times post-transfection. Data are shown as average and standard deviation of two experiments performed in triplicate (N = 6). Significance (Student’s T-test): ***p<0.0005; *p<0.05.

Next, we analyzed the effect of treatment with Gua, Ade, Cyt, and Uri in a surrogate single cycle infection model, taking advantage of spread-deficient bona fide HCV virions bearing a luciferase reporter gene (trans-complemented HCV particles, HCVtcp). This system recapitulates early stages of the infection including viral entry, primary translation and genome replication, overall efficiency of which is proportional to reporter gene activity [12]. The results (Fig 2A) show that a selective HCV entry inhibitor, hydroxyzine (HDH), strongly interfered with reporter gene accumulation, as previously documented [13] (Fig 2A). Of the four natural nucleosides, only Gua exerted a significant inhibitory role, as shown by reduced luciferase levels in these cells (Fig 2A), and suggesting that an early step of the infection preceding viral assembly is significantly inhibited by Gua. To further dissect the impact of Gua on HCV replication, we analyzed the effect of Gua, Ade, Cyt and Uri treatment at different times in the replication of a dicistronic subgenomic genotype 2a (JFH-1) replicon bearing a luciferase reporter gene [12]. The objective was to analyze if the effect took place at the level of IRES-dependent translation (5 h post-transfection) or during RNA replication (24 and 48 h post-transfection) [13]. The results (Fig 2B) show that there are no differences among treatments at 5 h post-transfection, which excludes an effect on HCV IRES-dependent RNA translation or any spurious interference with reporter gene expression. However, Gua-treated cells showed a statistically significant 12- and 5-fold reduction in RNA replication at 24 and 48 h post-transfection respectively (Fig 2B). A modest (about 2-fold) but significant reduction was also observed in Ade-treated cells. However, treatment with Ade did not affect HCVtcp infection (Fig 2A) suggesting that only Gua significantly affects HCV infection by interfering with viral RNA replication, viral entry, and primary translation.

### Effect of Gua on a high fitness HCV population

The HCV p100 virus [HCV p0 passaged 100 times in Huh-7.5 reporter cells], shows a relative fitness that is 2.2 times higher than that of the HCV p0 parental population [14]. Since viral fitness can influence the response of the virus to antiviral agents [15–17], HCV p100 was used to study the response of a high fitness HCV to Gua. For this, HCV p100 was subjected to 5 serial passages in Huh-7.5 reporter cells using an initial m.o.i. of 0.05 TCID_50_ per cell, both in the absence and in the presence of Gua 500 or 800 μM. The results show a sustained drop in infectivity of 15 and 736 times along the passages as a result of treatment with 500 and 800 μM Gua, respectively. However, no decrease of infectivity below the limit of detection was observed throughout the five passages in any of the replicates (Fig 1E). Only the decrease in progeny production in the presence of 800 μM was statistically significant (Fig 1E). Thus, the results show increased resistance of HCV p100 to Gua compared to HCV p0 (Fig 1A, 1B and 1E) as previously observed with several antiviral drugs [16,17].

### Effect of Gua on the replication of other RNA viruses

To determine the specificity of the antiviral action exerted by Gua on HCV, and to rule out nonspecific effects that could affect any virus, comparative experiments were conducted with foot-and-mouth disease virus (FMDV), lymphocytic choriomeningitis virus (LCMV), and vesicular stomatitis virus (VSV). First, the CC_50_ values of Gua, Ade, Cyt, and Uri were determined for BHK-21 cells, as described in Materials and Methods. The values obtained (Table 2) indicate no detectable cytotoxicity of Gua, Cyt and Uri, and a CC_50_ value of 391 ± 68 μM for Ade. To determine the IC_50_ values of these nucleosides, BHK-21 cells were infected with FMDV, LCMV, and VSV, at an initial m.o.i. of 0.05 TCDI_50_ per cell in the presence of increased nucleoside concentrations and the production of infectious progeny was measured. The values obtained (Table 2) show that all nucleosides lacked inhibitory profile for FMDV. In contrast, purines were inhibitory for VSV, while all nucleosides were inhibitory for LCMV. However, the IC_50_ values were very high and the therapeutic indexes (TI) were consequently low (Table 2).

**Table 2:** Effects of nucleosides on BHK-21 cells viability and VSV, FMDV, and LCMV replication. CC_50_, IC_50_, and therapeutic index (TI, CC_50_/IC_50_) values are shown for Adenosine (Ade), Cytidine (Cyt), Guanosine (Gua), and Uridine (Uri) in BHK-21 cells.

		IC_50_ (μM) (TI)		
	CC_50_ (μM)	VSV	FMDV	LCMV
Ade	391 ± 68	650 ± 105.4 (0.6)	> 800 (n.d.)	213 ± 56.2 (1.8)
Cyt	> 800	> 800 (n.d.)	> 800 (n.d.)	566.4 ± 216.7 (≥ 1.4)
Gua	> 800	734 ± 59.4 (≥ 1.1)	> 800 (n.d.)	348 ± 36 (≥ 2.3)
Uri	> 800	> 800 (n.d.)	> 800 (n.d.)	157.8 ± 7.6 (≥ 5.1)

n.d. Not Determined.

**Fig 3:**
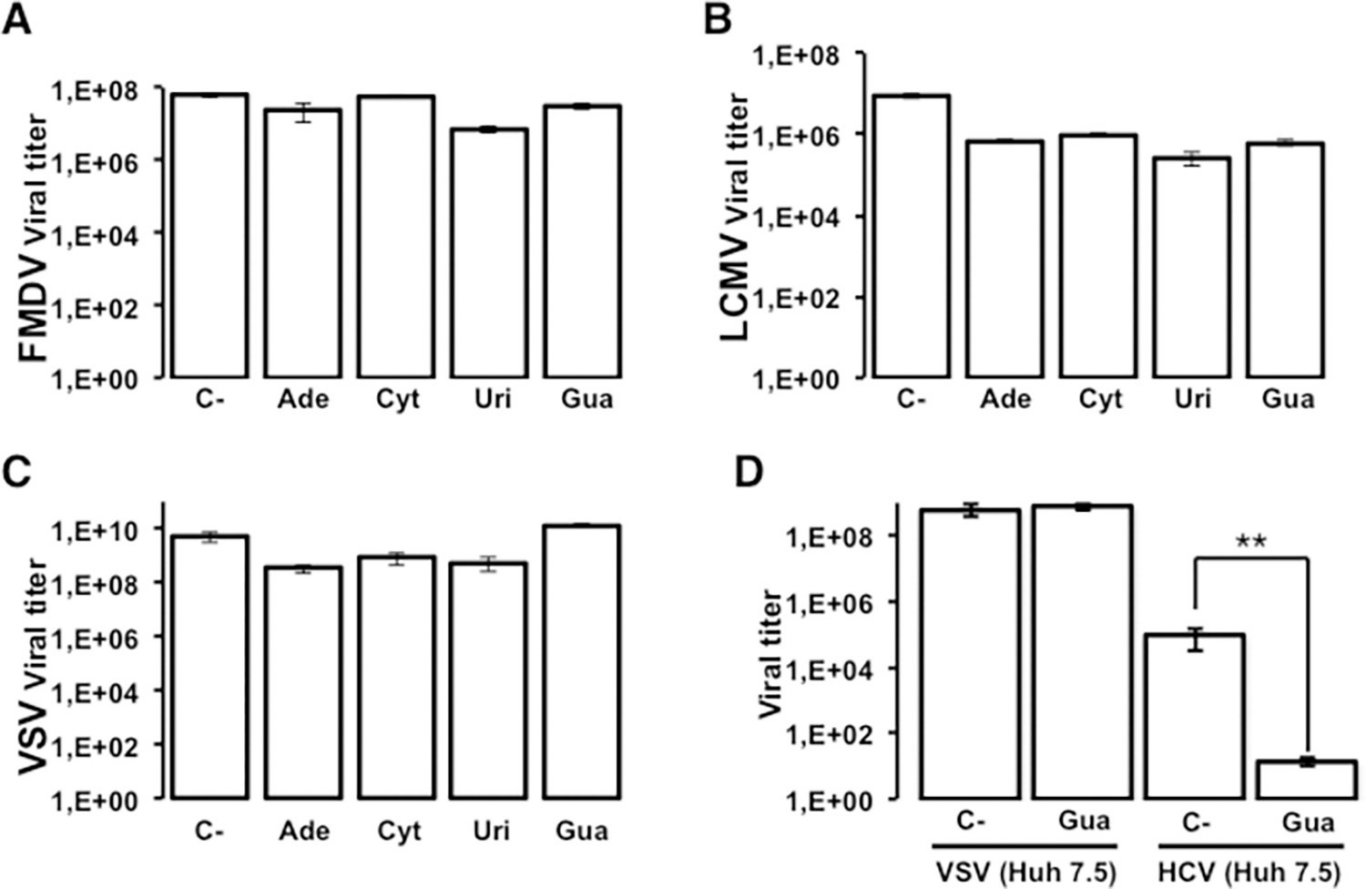
Effect of nucleosides on FMDV, LCMV and VSV replication. Effect of adenosine (Ade), cytidine (Cyt), uridine (Uri), and guanosine (Gua) on the production of infectious progeny of FMDV **(A)**, LCMV **(B)** and VSV **(C)** in BHK-21 cells at an initial m.o.i. of 0.05 TCID_50_ per cell, in the absence (C-) or presence of 800 μM of the indicated nucleoside. Infectivity was determined at passage 3 in the cell culture supernatant as described in Materials and Methods. **(D)** Comparative inhibition of VSV and HCV p0 progeny production in Huh-7.5 reporter cells in the presence of 800 μM Gua. The titer shown for HCV is the average (four replicas) of titers determined at passage 3 in the supernatants of the serial infections (corresponding to Fig 1). Procedures for serial infections and titration of infectivity are detailed in Materials and Methods. Significance (Student’s T-test): ** p < 0.005.

As an additional control for the specificity of HCV inhibition by Gua, the response of VSV, FMDV and LCMV to nucleoside treatment in serial infections was studied. BHK-21 cells were infected with FMDV, LCMV, and VSV with an initial m.o.i. of 0.05 TCDI_50_ per cell, and were subjected to 3 passages both in the absence and presence of nucleosides at a final concentration of 800 μM. The analysis of the viral populations in passage 3 showed no statistically significant difference from the viral titer obtained in the absence of treatment (Fig 3A–3C). Therefore, the results show that the only differences found were those of HCV treatment with Gua at 800 μM (Fig 1A and 1B). The presence of Gua did not affect the kinetics of FMDV, VSV or LCMV progeny production in serial infections, and the decrease of progeny production was less than one logarithm, regardless of the infection conditions and the final titer obtained (Figs 3 and S2). In contrast, Gua evoked a four-logarithm decrease in the infectious progeny production of HCV (Fig 3). Finally, to rule out that the inhibitory effect of Gua on HCV was solely due to the action of the nucleoside on the human hepatoma cells used in the experiments, we examined the production of VSV viral progeny in Huh-7.5 reporter cells which this virus also productively infects. High viral titers in the presence of 800 μM Gua were obtained for VSV, confirming a lack of antiviral activity of Gua against this virus also in Huh-7.5 reporter cells (Fig 3D).

### Effect of guanosine on HCV NS5B activity

**Fig 4:**
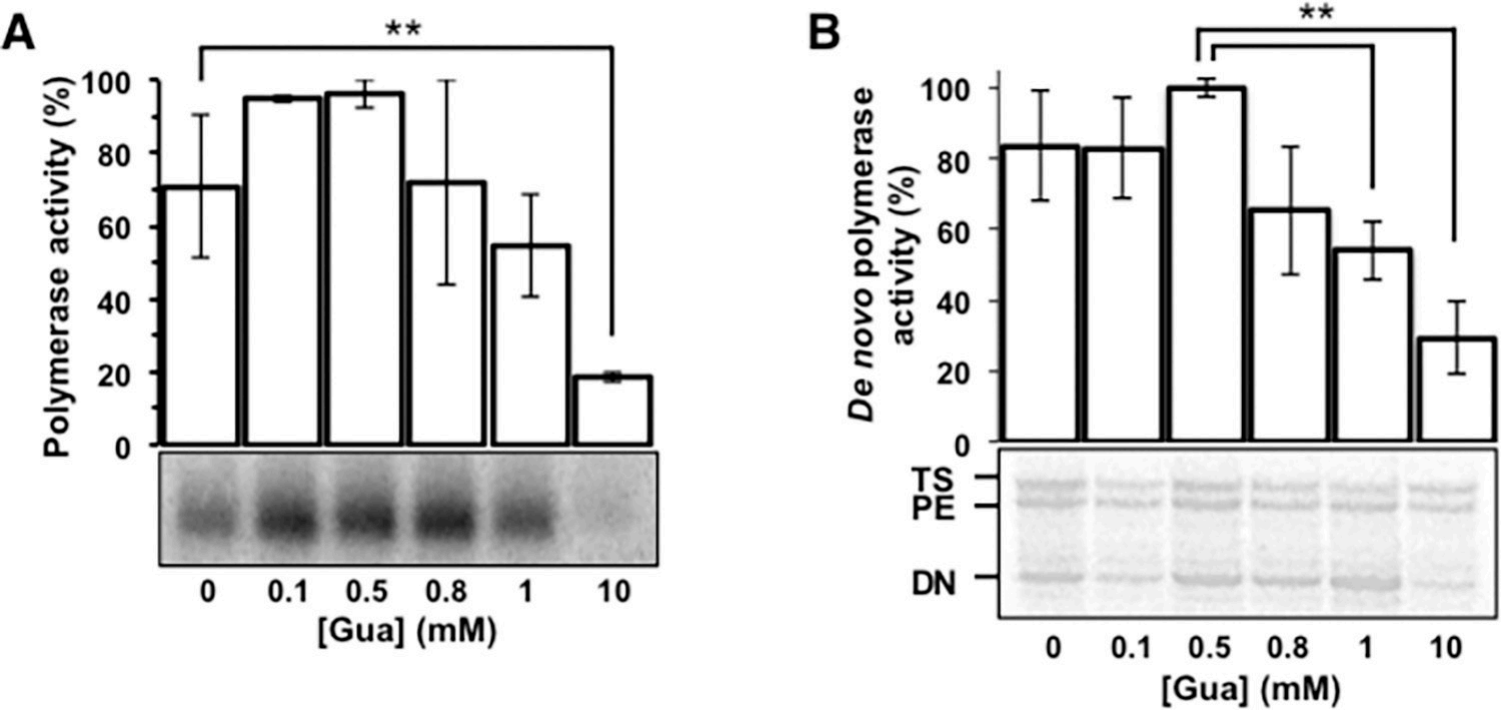
Effect of Gua on NS5BΔ21 RNA polymerase activity. **(A)** Recombinant HCV NS5B**Δ**21 polymerase was added to a reaction containing a 540-nt RNA template [18], the four nucleoside-triphosphates (ATP, CTP, GTP, and UTP) and the indicated concentrations of Gua. Product quantification from three replicates (average ± SEM) and a representative experiment (below) are shown. Polymerase activity is normalized with respect to its maximum activity. The band indicates a new synthesis RNA product of 540 nt length. **(B)** A representative experiment as in A, but using the 19-nt LE19 RNA as a template. DN, PE, and TS indicate reaction products of *de novo* synthesis, primer extension, and template switching, respectively [53]. Procedures are detailed in Materials and Methods. Significance (Student’s T-test): ** p < 0.005.

To analyze the mechanism by which Gua inhibits HCV replication we tested the effect of increasing Gua concentrations on HCV polymerase activity *in vitro*. A 570 nt RNA fragment corresponding to the E1/E2 region of the HCV genome [18] was replicated by HCV recombinant NS5B**Δ**21 in the presence of 100 μM ATP, CTP, GTP, and UTP, and at increasing concentrations of Gua (Fig 4A). NS5B polymerase activity increased with Gua concentration up to 500 μM. Even at 1 mM Gua, the RNA polymerase activity was similar to that obtained in absence of Gua. Only at very high Gua concentration (10 mM) the RNA polymerase activity showed a significant reduction (Fig 4A). Similar results were obtained using the 19-mer oligonucleotide LE19 (Fig 4B). Therefore, according to this *in vitro* RNA synthesis assay, the inhibition of HCV progeny production by Gua cannot be attributed to direct inhibition of the HCV RNA polymerase.

### Effect of guanosine on intracellular nucleotide pools

**Fig 5:**
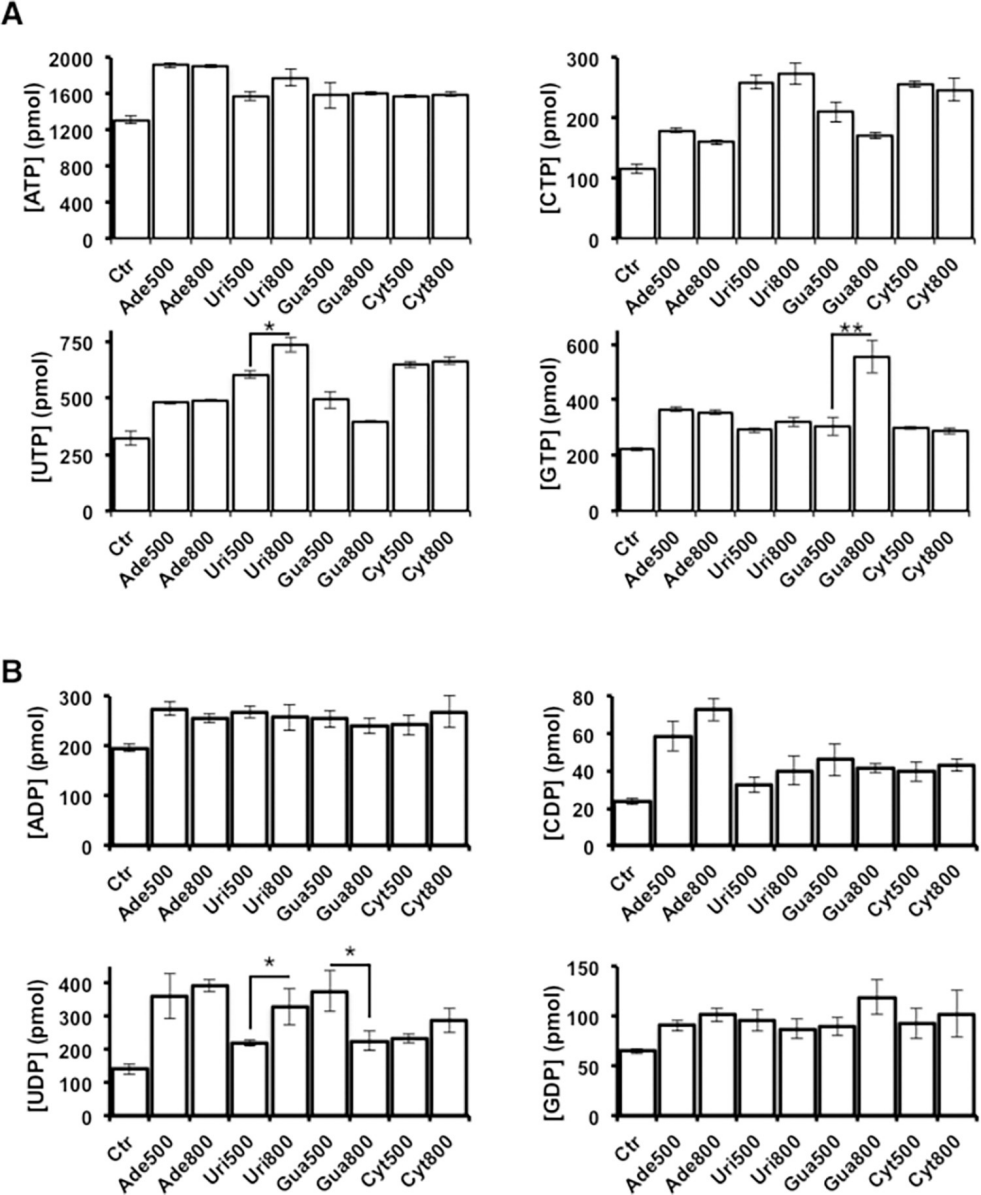
Effect of Gua on intracellular nucleotides. Effect of treatment of Huh-7.5 cells with 500 μM and 800 μM of Ade, Uri, Gua, and Cyt on the level of intracellular nucleoside-triphosphates (**A**) and intracellular nucleoside-diphosphates (**B**). Quantification from three different runs of two biological replicates (geometric mean ± SEM) are shown. Significance (Student’s T-test): * p < 0.05; ** p < 0.005; *** p < 0.0005.

To investigate whether the inhibition of HCV replication described above could be related to alterations in intracellular concentrations of ribonucleoside di- and triphosphate, the level of NTP and NDP in Huh-7.5 reporter cells was determined both in the absence and after 72 h of treatment with 500 or 800 μM of Ade, Cyt, Gua or Uri. All treatments induced an increase in the concentration of the nucleoside di- and triphosphate (Fig 5). However, only Uri and Gua treatments induced a concentration-dependent increase in UTP and GTP respectively, with the effect of Gua treatment being the most intense and statistically significant (Fig 5A). Intracellular NDP concentrations are shown in Fig 5B. Only the UDP concentration exhibited small variations and all of them with low statistical significance. With these values we also calculate the variation in the ratio between NTP and NDP (Table 3).

**Table 3:** Intracellular NTP/NDP ratio values.

	UTP/UDP^a^	CTP/CDP	ATP/ADP	GTP/GDP
Control	2.2	4.8	6.5	3.3
Ade500	1.3	3.0	6.8	4.0
Ade800	1.2	2.2	7.3	3.4
Uri500	2.7	7.8	5.8	3.0
Uri800	2.2	6.7	5.9	3.6
Gua500	1.4	4.6	6.9	3.8
Gua800	1.7	4.1	6.5	5.0
Cyt500	2.7	6.3	6.4	3.2
Cyt800	2.3	4.5	5.8	2.8

^a^NTP and NDP concentration values are those from Fig 5.

### Mutational effects of guanosine

**Fig 6:**
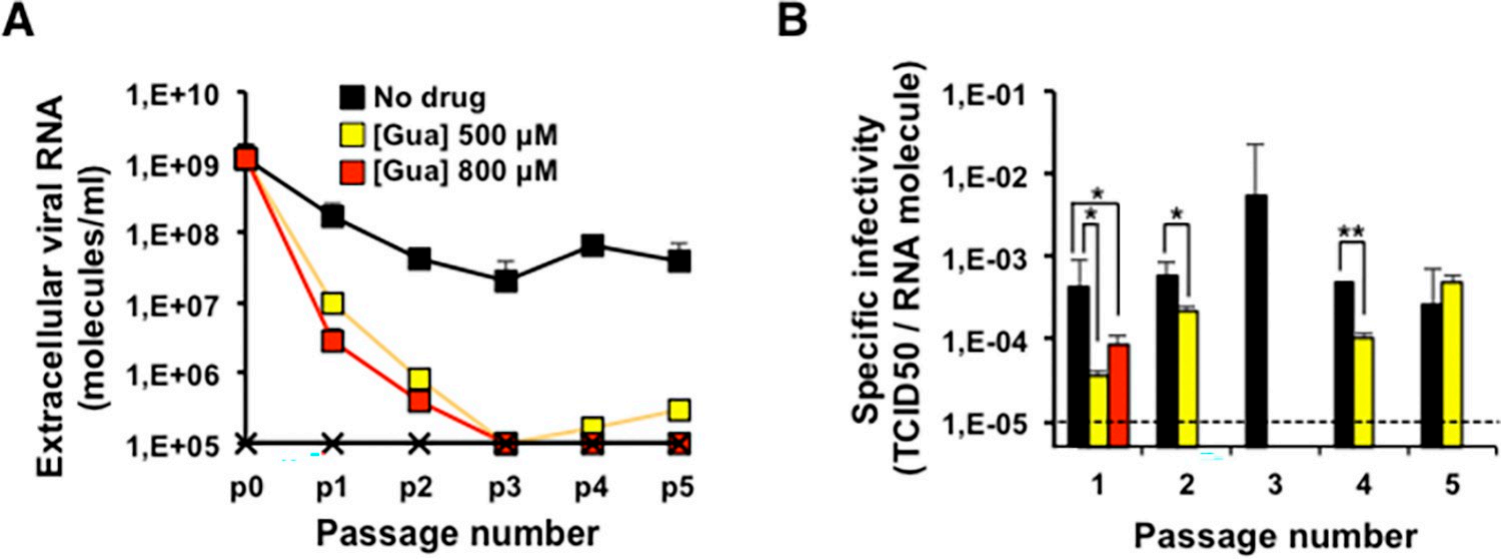
Effect of guanosine on HCV specific infectivity. Huh-7.5 reporter cells were infected with HCVp0 at an initial m.o.i. of 0.05 TCID50/cell, in the absence or presence of Gua at the indicated concentrations. HCV GNN infection was used as a negative control. **(A)** Extracellular viral RNA measured by quantitative RT-PCR in different passages. The populations correspond to those of the experiment described in Fig 1 and the values in each passage are the average of the three replicas; standard deviations are given. **(B)** Specific infectivities calculated from the infectivity values of the Fig 1A and 1B and the extracellular RNA concentrations indicated in Fig 8A. The horizontal dashed line indicates the limit of detection of viral RNA and specific infectivity. Black, yellow, and red symbols correspond to no drug, Gua 500 μM, and Gua 800 μM, respectively. Values for a HCV lethal mutant GNN (black crosses) are also shown. Details of the procedures are given in Materials and Methods. Significance (Student’s T-test): ** p < 0.005, * p < 0.05.

**Fig 7:**
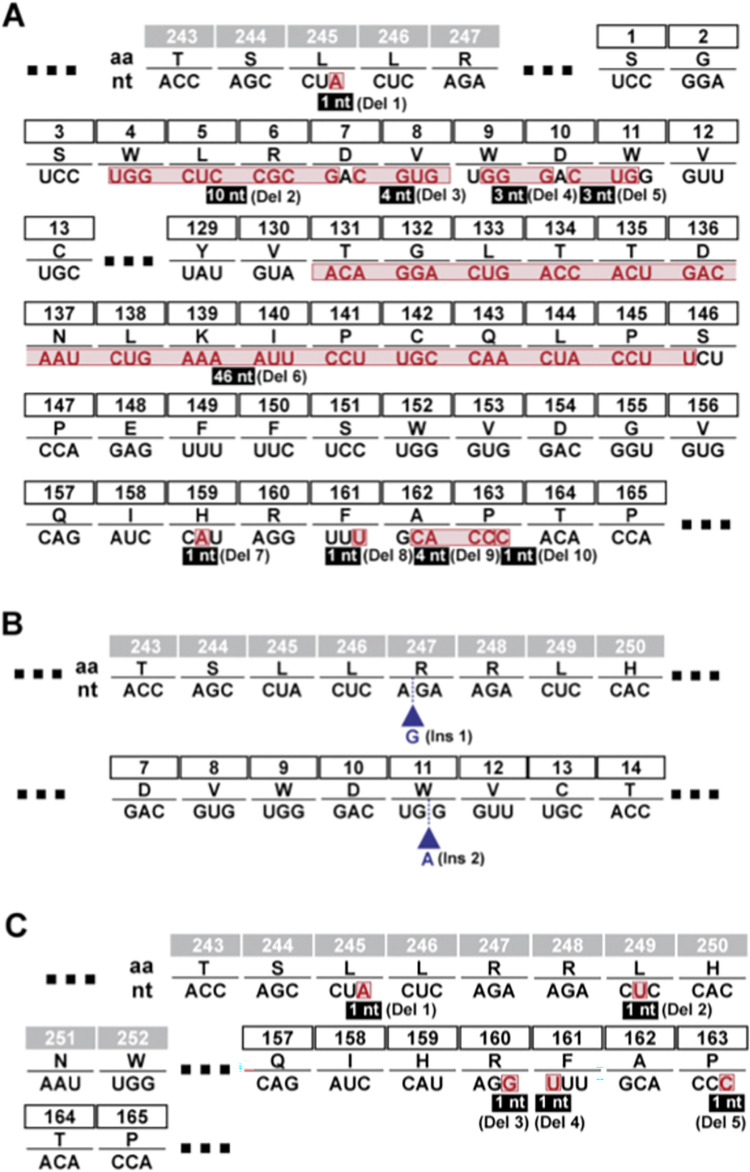
Indels found in the mutant spectrum of HCV p0 passaged in the presence of Gua. The nucleotide sequence of HCV genomic residues 6220 to 6758 was determined for 53 molecular clones derived from the population in absence of Gua, and 132 molecular clones from populations passaged in presence of Gua (data in Table 5). Deduced amino acids (single letter code) are given for residues located at the carboxy-terminal region of NS4B preceding NS5A amino acids. For clarity, only residues around insertions or deletions are shown; three squared points indicate missing amino acids (sequence is that of JFH-1; accession number AB047639). No indels were detected in the population passaged in absence of Gua. (**A**) Deletions in the population passaged in the presence of 500 μM guanosine. Red boxes indicate nucleotides that were deleted in a component of the mutant spectrum, with the deletion size indicated in the filled boxes. (**B**) Insertions in the mutant spectrum of the population passaged in the presence of 500 μM Gua are marked with a blue triangle. **(C)** Deletions found in the HCV populations passaged in the presence of 800 μM Gua. Procedures for HCV genome sequencing are described in Materials and Methods. The name of the indel as detailed in Table 5 is included next to the size.

To determine if Gua-related nucleotide pool effects were associated with the mutation repertoire exhibited by HCV during replication in Huh-7.5 cells, the mutant spectrum of the genomic region spanning the last 49 nucleotides of the NS4B gene and the first 490 nucleotides of the NS5A gene, was analyzed using molecular cloning and Sanger sequencing. Following three passages in absence or presence of Gua, the maximum mutation frequency resulted in a significant increase in the HCV populations passaged in the presence of Gua (p<0.0001 and p = 0.01 for Gua 500 μM, and Gua 800 μM, respectively; χ^2^ test) (Table 4). A hallmark of virus extinction by lethal mutagenesis is the decrease of specific infectivity (the ratio between viral infectivity and the amount of genomic viral RNA) [7]. Extinction by Gua occurred with a 2.8-fold to 11.8-fold decrease of specific infectivity in the first two passages in the presence of the compared drug, as quantified by infectivity and viral RNA in samples of the cell culture supernatants (Fig 6), suggesting that an increase in polymerase error rate was involved. The most remarkable change was that replication in the presence of Gua increased significantly the number of indels in the heteropolymeric genomic regions of the mutant spectrum (Table 5). Indels in homopolymeric regions ─consisting of at least three successive identical nucleotides─ were not considered because control experiments revealed that they can be amplification artifacts [19]. No indels were detected in the 53 molecular clones derived from the population passaged in the absence of Gua. In sharp contrast, 10 deletions and 2 insertions were present in the 64 molecular clones retrieved from the population passaged in the presence of 500 μM Gua, and 5 deletions in 68 molecular clones from the population passaged in the presence of 800 μM Gua (Table 5). The difference in the number of deletions is highly significant for the populations passaged in the presence of 500 μM and 800 μM Gua (p<0.001; test χ2). The higher number of indels in the population passaged in the presence of 500 μM Gua compared to the one passaged in the presence of 800 μM Gua can be explained as a result of the decreased viral population complexity as shown by a lower minimum mutation frequency and Shannon entropy (Table 4). The size of the deletions ranged from 1 to 46 nucleotides, some were found in a single clone, others in several clones, and none of the deletions and insertions were in phase; they all generated a premature STOP codon (Table 5 and Fig 7). All deletions of more than one nucleotide except deletion number 6 (17 of 18 deletions), had a G at the 3’ end of the positive strand and/or a G at the 3’ end of the negative strand (Figs 7 and S1). No other sequence patterns were detected. Therefore, the anti-HCV effect of Gua, exerted via nucleotide-mediated alterations of polymerase activity, is associated with the generation of multiple deletions during HCV replication.

**Table 4:** Mutant spectrum analysis of the hepatitis C virus populations passaged in the absence and presence of guanosine (Gua).

HCV population^a^	N. of nucleotides analyzed (clones/haplotypes)^b^	N. of different (total) mutations^c^	Mutation frequency		Normalized Shannon entropy (H_SN_)
			Minimum^d^	Maximum^e^	
HCV p0, [no drug] p3	28,208 (53/21)	23 (25)	8.2 x 10^−4^	8.9 x 10^−4^	0.5
HCV p0, [Gua 500 μM] p3	33,339 (64/34)	45 (79)	1.4 x 10^−3^	2.4 x 10^−3^	0.7
HCV p0, [Gua 800 μM] p3	35,606 (68/30)	32 (59)	9.0 x 10^−4^	1.7 x 10^−3^	0.6

^a^The populations analyzed are those schematically represented in Fig 1, and their origin is detailed in Materials and Methods; [no drug] means passages in absence of drug; [Gua 500 μM] means passages in the presence of 500 μM guanosine; [Gua 800 μM] means passages in the presence of 800 μM guanosine; p indicates passage number.

^b^The genomic region analyzed by molecular cloning-Sanger sequencing spans residues 6220 to 6758 of the NS4B- and NS5A-coding region; the residue numbering corresponds to the JFH-1 genome (GenBank accession number #AB047639). The values in parenthesis indicate the number of clones analyzed followed by the number of haplotypes (number of different RNA sequences) found in the mutant spectrum.

^c^Number of different and total mutations identified by comparing the sequence of each individual clone with the consensus sequence of the corresponding population.

^d^Data represent the average number of different mutations (counted relative to the consensus sequence of the corresponding population) per nucleotide in the components of the mutant spectrum. The statistical significance of the differences between two populations (χ^2^ test) is the following: HCV p0, [no drug] p3 versus HCV p0, [Gua 500 μM] p3: p = 0.0622; HCV p0, [no drug] p3 versus HCV p0, [Gua 800 μM] p3: p = 0.8257; HCV p0, [Gua 500 μM] p3 versus HCV p0, [Gua 800 μM] p3: p = 0.0977.

^e^Data represent the average number of total mutations (counted relative to the consensus sequence of the corresponding population) per nucleotide in the components of the mutant spectrum relative to the consensus sequence of the corresponding population. The statistical significance of the differences between two populations (proportion test) is the following: HCV p0, [no drug] p3 versus HCV p0, [Gua 500 μM] p3: p < 0.0001; HCV p0, [no drug] p3 versus HCV p0, [Gua 800 μM] p3: p = 0.0107; HCV p0, [Gua 500 μM] p3 versus HCV p0, [Gua 800 μM] p3: p = 0.0452

^f^The normalized Shannon entropy (H_SN_) was calculated as H_SN_(p) = $-\sum_{i=1}^{N}\;[p_{i}\cdot \ln\;p_{i}]/\ln\;N$, where p_i_ is the frequency of each sequence of the quasispecies and N is the number of genomes compared.

**Table 5:** Indels found in the mutant spectra of HCV p0 after 3 passages in the absence and presence of Gua 500 μM and 800 μM.

HCV population^a^	Indel	Position^c^	Number of deleted or inserted nucleotides	Region	Premature STOP position	# of clones
HCV p0, no Gua p3	**-**	0	-	-	-	-
HCV p0, [Gua 500 μM] p3	**Deletion 1**	6220	1	NS4B	6243	2
	**Deletion 2** ^**b**^	6278–6287	10	NS5A	6315	4
	**Deletion 3** ^**b**^	6289–6292	4	NS5A	6315	4
	**Deletion 4** ^**b**^	6294–6296	3	NS5A	6315	4
	**Deletion 5** ^**b**^	6298–6300	3	NS5A	6315	4
	**Deletion 6**	6659–6704	46	NS5A	6864	1
	**Deletion 7**	6744	1	NS5A	6864	1
	**Deletion 8**	6751	1	NS5A	6864	1
	**Deletion 9**	6753–6756	4	NS5A	6864	1
	**Deletion 10**	6757	1	NS5A	6864	1
	**Insertion 1**	6225	1	NS4B	6246	1
	**Insertion 2**	6301	1	NS5A	6298	1
HCV p0, [Gua 800 μM] p3	**Deletion 1**	6220	1	NS4B	6243	13
	**Deletion 2**	6231	1	NS4B	6243	1
	**Deletion 3**	6748	1	NS5A	6864	3
	**Deletion 4**	6749	1	NS5A	6864	1
	**Deletion 5**	6757	1	NS5A	6864	2

^a^The populations analyzed are those schematically represented in Fig 1A and 1B, and their origin is detailed in Materials and Methods; [Gua 500 μM] means passages in the presence of 500 μM guanosine; [Gua 800 μM] means passages in the presence of 800 μM guanosine; p indicates passage number.

^b^Deletions 2, 3, 4 and 5 were found always together.

^c^The genomic region analyzed by molecular cloning-Sanger sequencing spans residues 6220 to 6758 of the NS4B- and NS5A-coding region; the residue numbering corresponds to the JFH-1 genome (GenBank accession number #AB047639).

### Effect of nucleoside di- and triphosphate imbalance on HCV NS5B activity *in vitro*

**Fig 8:**
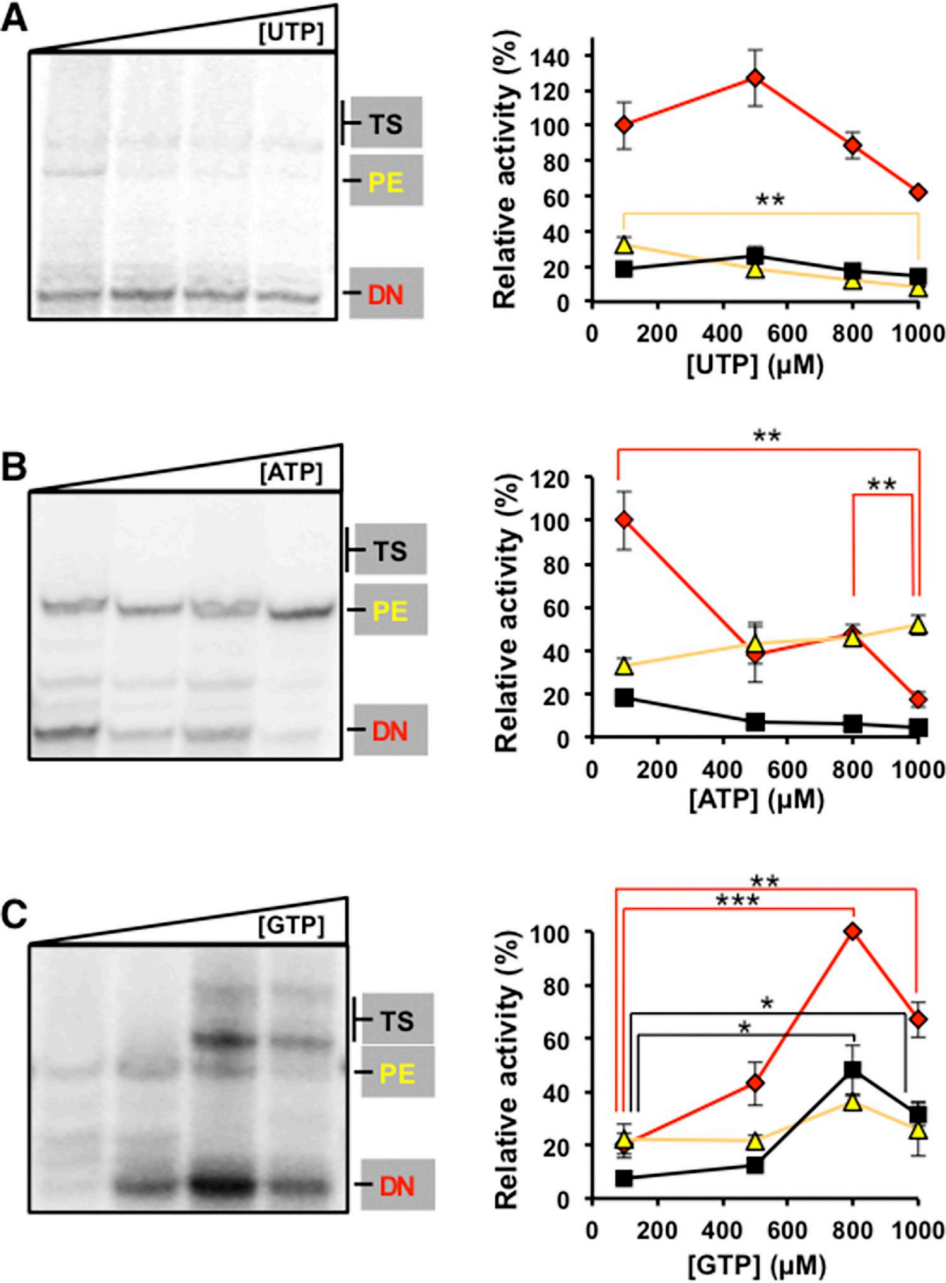
Effect of nucleoside-triphosphate concentration on NS5BΔ21 RNA polymerase activity. (**A)** Polyacrylamide gel showing the products for de novo (DN), primer extension (PE) and template switching (TS) obtained with HCV NS5BΔ21 at increasing concentrations (100, 500, 800, and 1000 μM) of UTP in the presence of radiolabeled α^32^P-CTP. ATP and GTP concentrations were maintained at 100 μM and 500 μM, respectively (left panel). Graphic representation of densitometric values obtained from the electropherogram shown in A (red diamonds, yellow triangles, and black squares correspond to *de novo* (DN), primer extension (PE), and template switching (TS) activities, respectively) (right panel). **(B)** Corresponds to experiments as in **A** but for increasing concentrations of ATP, with UTP and GTP maintained at 100 μM and 500 μM, respectively. **(C)** Corresponds to experiments as in **A** but for increasing concentrations of GTP, with ATP and UTP both maintained at 100 μM. Activities were normalized to their maximum values. Densitometric data represent the mean of at least three independent experiments. Error bars correspond to standard error of the mean. Horizontal lines indicate statistically significant differences (Student’s T-test) between the activity values that link, using the same color code as the activity type. Details of the activity measurements are given in Materials and Methods. Significance (Student’s T-test): *** p < 0.0005, ** p < 0.005, * p < 0.05.

To explore if changes in nucleotide concentrations might affect HCV polymerase activity, we performed *in vitro* RNA polymerization experiments with recombinant NS5B**Δ**21 in the presence of increasing concentrations of NTPs or NDPs. CTP and CDP were not included in the analyses because they showed small intracellular variations (Fig 5) and CTP was chosen as the carrier of the radioisotope. *De novo* (DN), primer extension (PE) and template switching (TS) polymerase activities were measured in the presence of increasing concentrations of the corresponding triphosphate nucleosides (Fig 8). This experiment replicates the procedures used in previous studies carried out to establish the best experimental conditions with this template [20,21]. A high UTP concentration of 1 mM slightly but significantly decreased primer extension activity (Fig 8A). However, the main effect of NTP concentration was on the *de novo* RNA synthesis, with a significant decrease at high ATP concentration (Fig 8B), and a significant increase at high GTP concentration (Fig 8C). The enhance in the *de novo* RNA synthesis was accompanied by an increase of template switching (Fig 8C). The effect observed by increasing the concentration of GTP was described previously [20,21] and is due to the incorporation of this nucleotide only in the first position during the *de novo* synthesis process with this system. This experiment allowed establishing the concentration of 500 μM GTP and 100 μM ATP, and UTP as the most suitable for the study of RNA-polymerase activity with this system.

**Fig 9:**
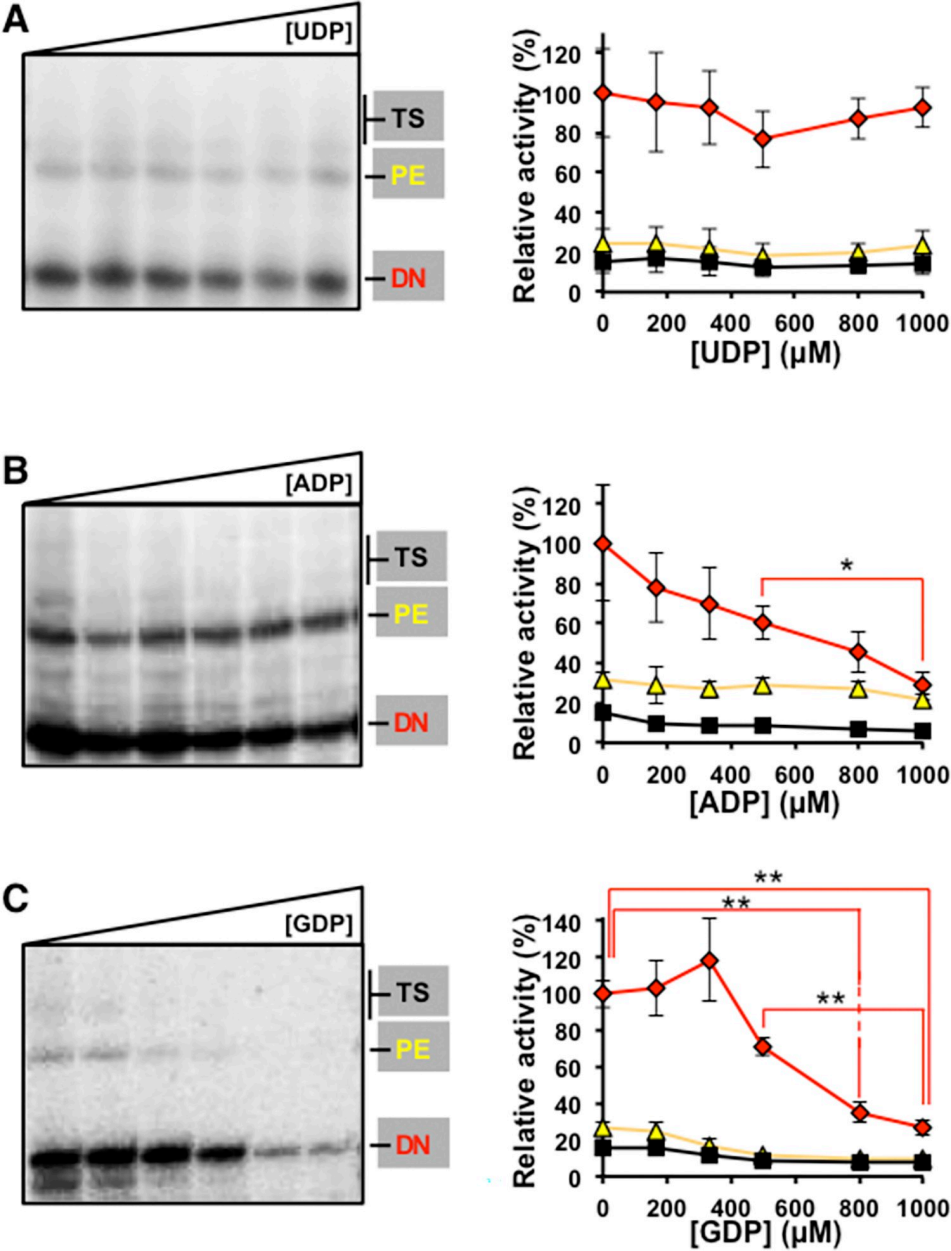
Effect of NDPs on NS5BΔ21 RNA polymerase activity. **(A)** Polyacrylamide gel showing the products for *de novo* (DN), primer extension (PE) and template switching (TS) obtained with HCV NS5BΔ21 at increasing concentrations (0, 166, 333, 500, 800, and 1000 μM) of UDP in the presence of ATP and UTP at a final concentration of 100 μM, GTP at 500 μM, and radiolabeled α^32^P-CTP (left panel). Graphic representation of densitometric values obtained from the electropherogram shown in A (red diamonds, yellow triangles, and black squares correspond to *de novo* (DN), primer extension (PE), and template switching (TS) activities, respectively) (right panel). **(B)** Corresponds to experiments as in **A** but for increasing concentrations of ADP. **(C)** corresponds to experiments as in **A** but for increasing concentrations of GDP. Activities were normalized to their maximum values. Densitometric data represent the mean of at least three independent experiments. Error bars correspond to standard error of the mean. Horizontal lines indicate statistically significant differences (Student’s T-test) between activity values, using the same color code as the activity type. Details of the activity measurements are given in Materials and Methods. Significance (Student’s T-test): ** p < 0.005, * p < 0.05.

Since increasing concentrations of Gua also altered the intracellular NDP concentrations (Fig 5), we investigated if the presence of increasing concentration of NDPs might affect the NS5B RNA polymerase activity *in vitro*. DN, PE, TS activities were measured in the presence of increasing concentrations of the corresponding diphosphate nucleosides at a fixed nucleoside-triphosphate concentration (Fig 9). The main effect of the presence of NDP was on the *de novo* RNA synthesis, with a significant decrease at high ADP and GDP concentrations. Differences in primer extension and template switching was not statistical significant (Fig 9).

## Discussion

Nucleoside derivatives are the most important family of drugs targeting viral polymerases, but the antiviral capacity of natural nucleosides has not been described [5]. Interestingly, we observed an inhibitory effect of Gua when it was used in experiments to analyze the impact of mycophenolic acid and ribavirin on HCV progeny production [11]. Here, we document inhibition of HCV replication by Gua in single and serial infections of Huh-7.5 cells that led to loss of infectivity without significant toxicity for the host cells. The antiviral action of Gua was also exerted on high fitness HCV, albeit without loss of infectivity after 5 passages in the presence of Gua, in agreement with the drug resistance phenotype displayed by high fitness HCV (Fig 1) [14–17]. The antiviral effect of Gua was not observed for FMDV, VSV or LCMV in BHK-21 cells, nor for VSV in Huh-7.5 cells (Figs 3 and S2).

RNA synthesis by NS5BΔ21 was not significantly affected by Gua concentrations up to 1 mM. Therefore, the inhibition of virus progeny production is unlikely to be the result of direct polymerase inhibition by Gua. This result is consistent with previous work that showed the ability of NS5B to initiate RNA synthesis with this nucleoside [20]. In contrast to Gua, altered intracellular nucleotide concentrations affected the activity of NS5B, in particular an alteration of the *de novo* RNA synthesis by the GTP/GDP and/or ATP/ADP balances (Fig 9). The NS5B protein has an allosteric binding site of GTP and the balance between NDP and NTP might modulate RNA synthesis through this site [21–23].

Some GTP-dependent proteins play a role in the HCV replication cycle. They include proteins involved in virus entry into the cells (i.e HRas), proteins involved in translation (i.e.the eIF5B factor), or in replication (i.e. GBF1) [24–26]. The GTP-dependent Rab18 protein, which is located in the lipid droplets, is involved in capturing proteins such as viral protein NS5A into the replication complex [27]. The increase in GTP concentration and in the GTP/GDP ratio observed in Gua-treated cells may affect the functionality of these proteins.

Some antiviral drugs exert their activity through alterations in the intracellular nucleotide pools [6,28]. Treatment of Huh-7.5 reporter cells with natural nucleosides produced an overall increase of intracellular nucleoside di- and tri-phosphate concentrations (Fig 5). However, only the cells treated with Gua significantly increased the intracellular concentration of GTP in a dose-dependent manner.

Intracellular concentration of mono- and di-phosphate nucleotides also modulates metabolic pathways critical for virus replication. For example, HCV proteins NS4B and NS5A inhibit the cellular protein AMP-activated protein kinase (AMPK) [29]. Inhibition of AMPK induces the synthesis of fatty acids and cholesterol that are of vital importance in the HCV replication sites. ADP activates AMPK [30,31], and the inactivation of AMPK by NS4B and NS5A is ineffective when ADP is increased [29]. As a result, there is no longer accumulation of fatty acids and cholesterol, and viral replication stops. Metformin activates AMPK by increasing AMP and ADP, and this effect has been associated with inhibition of HCV replication [30,32,33]. However, the increase in intracellular ADP concentration is small and independent of the treatment administered so this mechanism does not seem to be responsible for the inhibition of HCV replication. Whether ATP acts only as a substrate or it also exerts some regulatory role needs to be further analyzed.

Defective viral genomes are increasingly recognized as players in virus-host interactions (reviewed in [34]). They are associated with multiple types of genetic lesions ranging from point mutations to large deletions. Deletions can result from polymerase slippage over one or several nucleotides, but the environmental factors that may trigger their occurrence are unknown. Generation of RNA deletions has been documented during *in vitro* replicase copying of viral RNAs [35], and deletions have been observed in many viruses [34,36–39]. Template switching is considered as the primary mechanism of copy-choice recombination of poliovirus in cells [40], and the primary mechanism of poly(rU) RNA synthesis by poliovirus polymerase [41]. The observed increase of indels in the presence of high GTP concentrations may be linked to the enhanced template switching observed with the HCV polymerase *in vitro*. The finding of a G closing the deletion in 17 out of 18 deletions could be an indication that the *de novo* initiation site, the allosteric GTP binding site or both play an important role in the mechanism of production of defective viral genomes. Despite limited information on the origin of HCV defective genomes, there is solid evidence of the implication of defective viral genomes in the interference with replication of their standard infectious counterparts [42], including a contribution to viral extinction by lethal mutagenesis [43,44]. They also play a role as stimulators of antiviral responses [45], and as mediators of virus attenuation and persistence [39,46,47], among other functions [34].

Defective genomes have been described for HCV, including in-frame deletion mutants. They are present in patient plasma, exosomes and liver biopsies and they may play regulatory roles during viral replication [48–52]. Little is known about the molecular mechanisms of generation of defective genomes despite detailed accounts of their high m.o.i.-dependent selection based on molecular complementation with standard genomes [34]. Our results provide evidence of a mechanism of generation of defective HCV genomes fuelled by nucleoside pool effects on HCV polymerase activity. This is accompanied by a significant reduction of the specific infectivity of the passaged viral pools, demonstrating the increasing presence of non-infectious viral genomes in the supernatants of Gua-treated cells. In addition to unveiling a possible mechanism of generation of defective HCV genomes, our results open the possibility that the alteration of cellular metabolic pathways may be a complementary strategy to the action of antiviral agents to produce reductions in viral load and promote the extinction of HCV.

## Material and methods

### Reagents and plasmids

Nucleosides Ade, Cyt, Gua, and Uri, as well as nucleoside di- and tri-phosphates were purchased from Sigma-Aldrich. Plasmid pNS5BΔ21 encoding the HCV NS5B that lacks the C-terminal 21 hydrophobic amino acids to enhance solubility has been described previously [53]. The resulting expression vector allows the expression of a tagged NS5BΔ21 with six histidine residues at its C terminus to aid in protein purification.

### Cells and viruses

The origin of Huh 7.5, Huh 7-Lunet, Huh-7.5 reporter cell lines and procedures used for cell growth in Dulbecco’s modification of Eagle’s medium (DMEM), have been described [11]. Cell lines were incubated at 37°C and 5% CO_2_. We used the following viruses in the experiments: HCV p0, obtained from HCVcc [Jc1FLAG2(p7-nsGluc2A)] (genotype 2a) and GNN [GNNFLAG2(p7-nsGluc2A)] (a replication-defective virus with a mutation in the NS5B RNA-dependent RNA polymerase) [11,54]. Mock-infected cells maintained in parallel with the infected cultures were prepared to control the absence of contaminations; no infectivity in the mock-infected cultures was identified in the experiments.

Trans-encapsidated HCV virions (HCVtcp) were produced by electroporation into packaging cells of a subgenomic, dicistronic HCV replicon bearing a luciferase gene, as previously described [12]. Supernatants of the electroporated cells were titrated to determine the optimal dose rendering detectable luciferase activity at 48 hours post-inoculation. The same subgenomic replicon was used for lipofection experiments, using lipofectamine2000 transfection reagent as previously described [13].

### Production of viral progeny and titration of infectivity

The procedures used to obtain the initial virus HCV p0 and for serial infections of the hepatoma Huh-7.5 reporter cells have been described [14]. Briefly, electroporation of Huh-7 Lunet cells was performed with 10 μg of the transcript of HCVcc (Jc1 or the negative control GNN) (260 volts, 950 μF). Then, electroporated cells were passaged every 3–4 days before cells became confluent; passages were continued until 30 days post-electroporation. Subsequently, the cell culture supernatants were pooled to concentrate the virus 20 times using 10,000 MWCO spin columns (Millipore), and aliquots were stored at −70°C [14]. For titration of HCV infectivity, cell culture supernatants were serially diluted and applied to Huh-7.5 cells. After 3 days post-infection the cell monolayers were washed with PBS, fixed with ice-cold methanol, and stained with anti-NS5A monoclonal antibody 9E10 [14].

### Toxicity test and inhibitory concentration

The CC_50_ was calculated by seeding 96-well plates with Huh-7.5 cells and exposing them to the compound under study during 72 hours. MTT [3-(4,5-dimethylthiazol-2-yl)-2,5-diphenyltetrazolium bromide] was added at 500 μg/ml; after 4 h crystals were dissolved with 100 μl of DMSO and the O.D. measured at 550 nm; 50% cytotoxicity was calculated from quadruplicate determinations [11].

IC_50_ values were calculated relative to the controls without treatment (defined as 100% infectivity) [55]. Determinations were performed in triplicate.

### RNA extraction, cDNA synthesis, PCR amplification, and nucleotide sequencing

Intracellular RNA was obtained from infected cells using the Qiagen RNeasy kit (Qiagen, Valencia, CA, USA). RNA from cell lysates or cell culture supernatants was extracted using the Qiagen QIAamp viral RNA mini kit (Qiagen, Valencia, CA, USA). Reverse transcription (RT) of different HCV genomic regions was performed using avian myeloblastosis virus (AMV) reverse transcriptase (Promega), and subsequent PCR amplification was carried out using AccuScript (Agilent Technologies), with specific primers. Primers for the HCV amplification and the sequencing have been described [11,14,17,19]. Agarose gel electrophoresis was used to analyze the amplification products, using HindIII-digested Φ-29 DNA as a molecular mass standard. In parallel, mixtures without template RNA were reverse transcribed and amplified to monitor the absence of cross-contamination by template nucleic acids. Nucleotide sequences of HCV RNA were determined on the two strands of the cDNA copies [11,54]; only mutations detected in the two strands were considered. To analyze the complexity of mutant spectra by molecular cloning and Sanger sequencing, HCV RNA was extracted and subjected to RT-PCR to amplify the NS5A-coding regions, as has been previously described [11]. Amplifications with template preparations diluted 1:10 and 1:100 were performed to ensure that an excess of template in the amplifications was used in the mutant spectrum analysis; the molecular cloning was performed from the undiluted template only when the 1:100-diluted template produced also a DNA band; this procedure avoids complexity biases due to redundant amplifications of the same initial RNA templates [11]. Control analyses to confirm that mutation frequencies were not affected by the basal error rate during amplification have been previously described [56]. Amplified DNA was ligated to the vector pGEM-T (Amersham) and used to transform *Escherichia coli* DH5α; individual colonies were taken for PCR amplification and nucleotide sequencing, as previously described [55].

### NDP and NTP pool analysis

The procedure used has been previously described [11]. Briefly, Huh-7.5 cells (2×10^6^ cells) were washed with PBS and incubated with 900 μl of 0.6 M trichloroacetic acid on ice for 10 min. A precooled mixture of 180 μl of Tri-n-octylamine (Sigma) and 720 μl of Uvasol (1,1,2-trichlorotrifluoroethane, Sigma) was added to the 900 μl extract, vortexed for 10 s, centrifuged 30 s at 12,000 × g at 4°C, and stored at −80°C prior to analysis. One hundred μl samples were applied to a Partisil 10 SAX analytical column (4.6 mm×250 mm) (Phenomenex) with a Partisil 10 SAX guard cartridge column (4.6×30 mm) (Phenomenex) using a Nexera-i HPLC system connected to a photodiode array detector (Shimazdu). NDP and NTP were separated at a eluent flow rate of 0.8 ml/min and detected with ultraviolet light at a wavelength of 254 nm. The column was pre-equilibrated with 60 ml of 7 mM NH_4_H_2_PO_4_, pH 3.8 (buffer A). The separation program started with 22.5 min of an isocratic period with buffer A, continued with a linear gradient of 112.5 min to the high concentration buffer 250 mM NH_4_H_2_PO_4_, 500 mM KCl, pH 4.5 (buffer B) and ended with an isocratic period of 37.5 min with buffer B. A processing method was done using the Lab Solutions chromatography software (Shimazdu Corporation). To this end, 50 μl of 20 pmol/μl UTP, CTP, ATP and GTP (Jena Bioscience), were separated prior to sample analysis. The HPLC analysis did not separate rNTPs from dNTPs, or rNDPs from dNDPs. However, since the absolute concentration of rNTP and rNDP is several orders of magnitude greater than that of dNTPs dNDPs, we consider the value obtained as the concentration of rNTPs and rNDPs. Determinations were carried out with two independent biological samples, each one in triplicate for NDPs and NTPs. The amount of each nucleoside in the cell extracts was normalized in relation to the peak corresponding to the NAD molecule obtained in the same run. NAD peak in the sample showed the same retention time and spectrum of pure NAD (Sigma) analyzed under the same chromatographic conditions.

### Quantification of HCV RNA

Real time quantitative RT-PCR was performed with the Light Cycler RNA Master SYBR Green I kit (Roche), following the manufacturer’s instructions, as previously described [14]. The 5′-UTR non-coding region of the HCV genome was amplified using as primers oligonucleotide HCV-5UTR-F2 (5′- TGAGGAACTACTGTCTTCACGCAGAAAG; sense orientation; the 5′ nucleotide corresponds to HCV genomic residue 47), and oligonucleotide HCV-5UTR-R2 (5′- TGCTCATGGTGCACGGTCTACGAG; antisense orientation; the 5′ nucleotide corresponds to HCV genomic residue 347). Quantification was relative to a standard curve obtained with known amounts of HCV RNA, obtained by *in vitro* transcription of plasmid GNN DNA [54]. Reaction specificity was monitored by determining the denaturation curve of the amplified DNAs. Mixture without template RNA and RNA from mock-infected cells were run in parallel to ascertain absence of contamination with undesired templates.

### NS5BΔ21 polymerase expression and purification

NS5B from strain pJ4-HC with a deletion of 21 aa at the C-terminal end (NS5B**Δ**21) was obtained as previously described [53,57]. This truncated protein displays polymerase activities that were not distinguished from those of the full-length enzyme [58]. Briefly, NS5B**Δ**21 was overexpressed in BL21DE3 Rosetta bacteria by IPTG induction and purified by affinity chromatography in a Ni-NTA column. Aliquots of the purest and most concentrated protein samples were adjusted to 50% glycerol and stored at -80°C until use. All purification processes were monitored by SDS-PAGE and Coomassie brilliant blue staining. Protein was quantified by SDS-PAGE gel imaging and protein determination using the Bradford assay.

### *In vitro* RdRP replication assays

RNA polymerase assays were carried out using two different RNA substrates, the symmetric substrate LE-19 (sequence 5’ UGUUAUAAUAAUUGUAUAC 3’), which is capable of primer-extension (PE), *de novo* initiation (DN), and template switching (TS) [53,57], and an RNA fragment encompassing HCV E1/E2 region (570 nt) [18]. Except when indicated otherwise, template RNA was pre-incubated for 15 minutes in a reaction mixture containing 20 mM MOPS, pH 7.3, 35 mM NaCl, 5 mM MnCl_2_, 100 nM NS5B and GTP at the indicated concentration for each experiment. Reactions were started by adding 1 μCi of α[^32^P]CTP (3000 Ci mmol, PerkinElmer Life Sciences) and nucleoside-triphosphate as indicated in each experiment. When appropriate, reactions were performed in the presence of increasing concentrations of nucleotide diphosphates. Reactions were carried out in a final volume of 10 μl, at room temperature for 30 minutes, and stopped using EDTA/formamide loading buffer. E1/E2 products were resolved using 1% agarose gel electrophoresis. Agarose gels were dried in an electrophoresis gel dryer (BioRad). LE19 products were resolved using denaturing polyacrylamide (23% PAA, 7 M urea) gel electrophoresis. Gels were exposed to phosphorimager screens and scanned with a Typhoon 9600 phosphorimager (Molecular Dynamics). Sample quantification was performed from parallel experiments. Band volume values were obtained by using the ImageQuant software provided with the apparatus (GE Healthcare).

### Statistical analyses

The statistical significance of differences between mutation frequencies was evaluated by the chi-square test. Statistical comparisons among groups were performed with Student’s T-tests, χ^2^ test, two-way ANOVA, and ANCOVA. Unless indicated otherwise, the statistical significance is indicated by asterisks: * p<0.05; ** p<0.005; *** p<0.0005.

## Supporting information

S1 Fig 5’ and 3’-end sequence alignment of the deletions of more than one nucleotide described in Fig 9. (TIF)

S2 Fig Effect of guanosine on viral progeny production of Vesicular Stomatitis Virus (VSV), Foot-and-mouth Disease Virus (FMDV) and Lymphocytic Choriomeningitis Virus (LCMV). BHK21c2 cells were seeded and 16 hours prior to infection cells were untreated or treated with 800 μM of guanosine. Cells were infected with two different m.o.i. (0.05 or 0.0005), supernatants were collected at different times after infection (0-, 2-, 5-, 8- and 24-hours post-infection) and viral progeny production was analyzed. The m.o.i. and the treatment used are indicated in the upper box. The viral titer expressed as log10 pfu/ml is indicated in the ordinate. The time post-infection is indicated in the abscissa expressed in hours. (**A**) Effect of the addition of 800 μM of guanosine on VSV infectious progeny production. The differences between the slope of the treated and untreated has been calculated using an ANCOVA test in GraphPad (m.o.i. 0.05 p-value = 0.86; m.o.i. 0.0005 p-value = 0.62). (**B, C**) Same as A but the virus used are FMDV (B) and LCMV (C). Statistical differences between slopes were not found for FMDV: (m.o.i. 0.05 p-value = 0.45; m.o.i. 0.0005 p-value = 0.26) and for LCMV: (m.o.i. 0.05 p-value = 0.58; m.o.i. 0.0005 p-value = 0.79). Titrations were carried out in triplicate.
(TIF)

## References

[1] Scheel TK, Rice CM. Understanding the hepatitis C virus life cycle paves the way for highly effective therapies. Nat Med. 2013;19(7):837–49. doi: 10.1038/nm.3248 ; PubMed Central PMCID: PMC3984536. 23836234 PMC3984536

[2] European Association for the Study of the Liver. Electronic address eee, European Association for the Study of the L. EASL Recommendations on Treatment of Hepatitis C 2018. J Hepatol. 2018;69(2):461–511. doi: 10.1016/j.jhep.2018.03.026 . 29650333

[3] Lombardi A, Mondelli MU, Hepatitis ESGfV. Hepatitis C: Is eradication possible? Liver Int. 2019;39(3):416–26. doi: 10.1111/liv.14011 . 30472772

[4] Hlaing NK, Banerjee D, Mitrani R, Arker SH, Win KS, Tun NL, et al. Hepatitis C virus therapy with peg-interferon and ribavirin in Myanmar: A resource-constrained country. World J Gastroenterol. 2016;22(43):9613–22. doi: 10.3748/wjg.v22.i43.9613 ; PubMed Central PMCID: PMC5116605. 27920482 PMC5116605

[5] De Clercq E, Li G. Approved Antiviral Drugs over the Past 50 Years. Clin Microbiol Rev. 2016;29(3):695–747. doi: 10.1128/CMR.00102-15 ; PubMed Central PMCID: PMC4978613. 27281742 PMC4978613

[6] Wray SK, Gilbert BE, Noall MW, Knight V. Mode of action of ribavirin: effect of nucleotide pool alterations on influenza virus ribonucleoprotein synthesis. Antiviral Res. 1985;5(1):29–37. doi: 10.1016/0166-3542(85)90012-9 . 3985606

[7] Perales C, Gallego I, de Avila AI, Soria ME, Gregori J, Quer J, et al. The increasing impact of lethal mutagenesis of viruses. Future Med Chem. 2019;11(13):1645–57. doi: 10.4155/fmc-2018-0457 . 31469331

[8] Asahina Y, Izumi N, Enomoto N, Uchihara M, Kurosaki M, Onuki Y, et al. Mutagenic effects of ribavirin and response to interferon/ribavirin combination therapy in chronic hepatitis C. Journal of hepatology. 2005;43(4):623–9. doi: 10.1016/j.jhep.2005.05.032 . 16098627

[9] Cuevas JM, Gonzalez-Candelas F, Moya A, Sanjuan R. Effect of ribavirin on the mutation rate and spectrum of hepatitis C virus in vivo. Journal of virology. 2009;83(11):5760–4. doi: 10.1128/JVI.00201-09 ; PubMed Central PMCID: PMC2681971. 19321623 PMC2681971

[10] Dietz J, Schelhorn SE, Fitting D, Mihm U, Susser S, Welker MW, et al. Deep sequencing reveals mutagenic effects of ribavirin during monotherapy of hepatitis C virus genotype 1-infected patients. Journal of virology. 2013;87(11):6172–81. doi: 10.1128/JVI.02778-12 ; PubMed Central PMCID: PMC3648094. 23536652 PMC3648094

[11] Ortega-Prieto AM, Sheldon J, Grande-Perez A, Tejero H, Gregori J, Quer J, et al. Extinction of hepatitis C virus by ribavirin in hepatoma cells involves lethal mutagenesis. PLoS One. 2013;8(8):e71039. doi: 10.1371/journal.pone.0071039 ; PubMed Central PMCID: PMC3745404. 23976977 PMC3745404

[12] Steinmann E, Brohm C, Kallis S, Bartenschlager R, Pietschmann T. Efficient trans-encapsidation of hepatitis C virus RNAs into infectious virus-like particles. Journal of virology. 2008;82(14):7034–46. doi: 10.1128/JVI.00118-08 ; PubMed Central PMCID: PMC2446957. 18480457 PMC2446957

[13] Mingorance L, Castro V, Avila-Perez G, Calvo G, Rodriguez MJ, Carrascosa JL, et al. Host phosphatidic acid phosphatase lipin1 is rate limiting for functional hepatitis C virus replicase complex formation. PLoS pathogens. 2018;14(9):e1007284. doi: 10.1371/journal.ppat.1007284 ; PubMed Central PMCID: PMC6161900. 30226904 PMC6161900

[14] Perales C, Beach NM, Gallego I, Soria ME, Quer J, Esteban JI, et al. Response of hepatitis C virus to long-term passage in the presence of alpha interferon: multiple mutations and a common phenotype. J Virol. 2013;87(13):7593–607. doi: 10.1128/JVI.02824-12 ; PubMed Central PMCID: PMC3700284. 23637397 PMC3700284

[15] Gallego I, Gregori J, Soria ME, Garcia-Crespo C, Garcia-Alvarez M, Gomez-Gonzalez A, et al. Resistance of high fitness hepatitis C virus to lethal mutagenesis. Virology. 2018;523:100–9. doi: 10.1016/j.virol.2018.07.030 . 30107298

[16] Gallego I, Sheldon J, Moreno E, Gregori J, Quer J, Esteban JI, et al. Barrier-Independent, Fitness-Associated Differences in Sofosbuvir Efficacy against Hepatitis C Virus. Antimicrob Agents Chemother. 2016;60(6):3786–93. doi: 10.1128/AAC.00581-16 ; PubMed Central PMCID: PMC4879421. 27067341 PMC4879421

[17] Sheldon J, Beach NM, Moreno E, Gallego I, Pineiro D, Martinez-Salas E, et al. Increased replicative fitness can lead to decreased drug sensitivity of hepatitis C virus. J Virol. 2014;88(20):12098–111. doi: 10.1128/JVI.01860-14 ; PubMed Central PMCID: PMC4178724. 25122776 PMC4178724

[18] Geller R, Estada U, Peris JB, Andreu I, Bou JV, Garijo R, et al. Highly heterogeneous mutation rates in the hepatitis C virus genome. Nat Microbiol. 2016;1(7):16045. doi: 10.1038/nmicrobiol.2016.45 . 27572964

[19] Moreno E, Gallego I, Gregori J, Lucia-Sanz A, Soria ME, Castro V, et al. Internal Disequilibria and Phenotypic Diversification during Replication of Hepatitis C Virus in a Noncoevolving Cellular Environment. Journal of virology. 2017;91(10):pii: e02505–16. doi: 10.1128/JVI.02505-16 ; PubMed Central PMCID: PMC5411618. 28275194 PMC5411618

[20] Ranjith-Kumar CT, Gutshall L, Kim MJ, Sarisky RT, Kao CC. Requirements for de novo initiation of RNA synthesis by recombinant flaviviral RNA-dependent RNA polymerases. J Virol. 2002;76(24):12526–36. doi: 10.1128/jvi.76.24.12526-12536.2002 ; PubMed Central PMCID: PMC136677. 12438578 PMC136677

[21] Ranjith-Kumar CT, Kim YC, Gutshall L, Silverman C, Khandekar S, Sarisky RT, et al. Mechanism of de novo initiation by the hepatitis C virus RNA-dependent RNA polymerase: role of divalent metals. J Virol. 2002;76(24):12513–25. doi: 10.1128/jvi.76.24.12513-12525.2002 ; PubMed Central PMCID: PMC136676. 12438577 PMC136676

[22] Cai Z, Yi M, Zhang C, Luo G. Mutagenesis analysis of the rGTP-specific binding site of hepatitis C virus RNA-dependent RNA polymerase. J Virol. 2005;79(18):11607–17. doi: 10.1128/JVI.79.18.11607-11617.2005 ; PubMed Central PMCID: PMC1212605. 16140738 PMC1212605

[23] Chinnaswamy S, Murali A, Li P, Fujisaki K, Kao CC. Regulation of de novo-initiated RNA synthesis in hepatitis C virus RNA-dependent RNA polymerase by intermolecular interactions. J Virol. 2010;84(12):5923–35. doi: 10.1128/JVI.02446-09 ; PubMed Central PMCID: PMC2876623. 20375156 PMC2876623

[24] Lebsir N, Goueslain L, Farhat R, Callens N, Dubuisson J, Jackson CL, et al. Functional and Physical Interaction between the Arf Activator GBF1 and Hepatitis C Virus NS3 Protein. J Virol. 2019;93(6). doi: 10.1128/JVI.01459-18 ; PubMed Central PMCID: PMC6401423. 30567983 PMC6401423

[25] Otto GA, Puglisi JD. The pathway of HCV IRES-mediated translation initiation. Cell. 2004;119(3):369–80. doi: 10.1016/j.cell.2004.09.038 . 15507208

[26] Zona L, Lupberger J, Sidahmed-Adrar N, Thumann C, Harris HJ, Barnes A, et al. HRas signal transduction promotes hepatitis C virus cell entry by triggering assembly of the host tetraspanin receptor complex. Cell Host Microbe. 2013;13(3):302–13. doi: 10.1016/j.chom.2013.02.006 . 23498955

[27] Salloum S, Wang H, Ferguson C, Parton RG, Tai AW. Rab18 binds to hepatitis C virus NS5A and promotes interaction between sites of viral replication and lipid droplets. PLoS Pathog. 2013;9(8):e1003513. doi: 10.1371/journal.ppat.1003513 ; PubMed Central PMCID: PMC3731246. 23935497 PMC3731246

[28] Cifuentes Kottkamp A, De Jesus E, Grande R, Brown JA, Jacobs AR, Lim JK, et al. Atovaquone Inhibits Arbovirus Replication through the Depletion of Intracellular Nucleotides. J Virol. 2019;93(11). doi: 10.1128/JVI.00389-19 ; PubMed Central PMCID: PMC6532098. 30894466 PMC6532098

[29] Mankouri J, Tedbury PR, Gretton S, Hughes ME, Griffin SD, Dallas ML, et al. Enhanced hepatitis C virus genome replication and lipid accumulation mediated by inhibition of AMP-activated protein kinase. Proceedings of the National Academy of Sciences of the United States of America. 2010;107(25):11549–54. doi: 10.1073/pnas.0912426107 ; PubMed Central PMCID: PMC2895084. 20534540 PMC2895084

[30] Ross FA, Jensen TE, Hardie DG. Differential regulation by AMP and ADP of AMPK complexes containing different gamma subunit isoforms. Biochem J. 2016;473(2):189–99. doi: 10.1042/BJ20150910 ; PubMed Central PMCID: PMC4700476. 26542978 PMC4700476

[31] Xiao B, Sanders MJ, Underwood E, Heath R, Mayer FV, Carmena D, et al. Structure of mammalian AMPK and its regulation by ADP. Nature. 2011;472(7342):230–3. doi: 10.1038/nature09932 ; PubMed Central PMCID: PMC3078618. 21399626 PMC3078618

[32] Rena G, Hardie DG, Pearson ER. The mechanisms of action of metformin. Diabetologia. 2017;60(9):1577–85. doi: 10.1007/s00125-017-4342-z ; PubMed Central PMCID: PMC5552828. 28776086 PMC5552828

[33] Huang H, Kang R, Wang J, Luo G, Yang W, Zhao Z. Hepatitis C virus inhibits AKT-tuberous sclerosis complex (TSC), the mechanistic target of rapamycin (MTOR) pathway, through endoplasmic reticulum stress to induce autophagy. Autophagy. 2013;9(2):175–95. doi: 10.4161/auto.22791 ; PubMed Central PMCID: PMC3552882. 23169238 PMC3552882

[34] Vignuzzi M, Lopez CB. Defective viral genomes are key drivers of the virus-host interaction. Nat Microbiol. 2019;4(7):1075–87. doi: 10.1038/s41564-019-0465-y . 31160826 PMC7097797

[35] Sabo DL, Domingo E, Bandle EF, Flavell RA, Weissmann C. A guanosine to adenosine transition in the 3’ terminal extracistronic region of bacteriophage Q beta RNA leading to loss of infectivity. Journal of molecular biology. 1977;112(2):235–52. doi: 10.1016/s0022-2836(77)80141-1 875018

[36] Davis AR, Hiti AL, Nayak DP. Influenza defective interfering viral RNA is formed by internal deletion of genomic RNA. Proceedings of the National Academy of Sciences of the United States of America. 1980;77(1):215–9. doi: 10.1073/pnas.77.1.215 PubMed Central PMCID: PMC348239. 6928614 PMC348239

[37] Nomoto A, Jacobson A, Lee YF, Dunn J, Wimmer E. Defective interfering particles of poliovirus: mapping of the deletion and evidence that the deletions in the genomes of DI(1), (2) and (3) are located in the same region. Journal of molecular biology. 1979;128(2):179–96. doi: 10.1016/0022-2836(79)90125-6 . 219204

[38] O’Hara PJ, Nichol ST, Horodyski FM, Holland JJ. Vesicular stomatitis virus defective interfering particles can contain extensive genomic sequence rearrangements and base substitutions. Cell. 1984;36(4):915–24. doi: 10.1016/0092-8674(84)90041-2 . 6323026

[39] Holland JJ, Villarreal LP. Persistent noncytocidal vesicular stomatitis virus infections mediated by defective T particles that suppress virion transcriptase. Proceedings of the National Academy of Sciences of the United States of America. 1974;71(8):2956–60. doi: 10.1073/pnas.71.8.2956 ; PubMed Central PMCID: PMC388597. 4370255 PMC388597

[40] Kirkegaard K, Baltimore D. The mechanism of RNA recombination in poliovirus. Cell. 1986;47(3):433–43. doi: 10.1016/0092-8674(86)90600-8 . 3021340 PMC7133339

[41] Arnold JJ, Cameron CE. Poliovirus RNA-dependent RNA polymerase (3Dpol) is sufficient for template switching in vitro. The Journal of biological chemistry. 1999;274(5):2706–16. doi: 10.1074/jbc.274.5.2706 . 9915801

[42] Roux L, Simon AE, Holland JJ. Effects of defective interfering viruses on virus replication and pathogenesis in vitro and in vivo. Advances in virus research. 1991;40:181–211. doi: 10.1016/s0065-3527(08)60279-1 . 1957718 PMC7131706

[43] Grande-Perez A, Lazaro E, Lowenstein P, Domingo E, Manrubia SC. Suppression of viral infectivity through lethal defection. Proceedings of the National Academy of Sciences of the United States of America. 2005;102(12):4448–52. doi: 10.1073/pnas.0408871102 ; PubMed Central PMCID: PMC555496. 15767582 PMC555496

[44] Martin V, Abia D, Domingo E, Grande-Perez A. An interfering activity against lymphocytic choriomeningitis virus replication associated with enhanced mutagenesis. The Journal of general virology. 2010;91(Pt 4):990–1003. doi: 10.1099/vir.0.017053-0 . 20007356

[45] Mims CA. Rift Valley Fever virus in mice. IV. Incomplete virus; its production and properties. British journal of experimental pathology. 1956;37(2):129–43. ; PubMed Central PMCID: PMC2082561. 13315888 PMC2082561

[46] Cave DR, Hendrickson FM, Huang AS. Defective interfering virus particles modulate virulence. Journal of virology. 1985;55(2):366–73. PubMed Central PMCID: PMC254942. doi: 10.1128/JVI.55.2.366-373.1985 2991562 PMC254942

[47] Santak M, Markusic M, Balija ML, Kopac SK, Jug R, Orvell C, et al. Accumulation of defective interfering viral particles in only a few passages in Vero cells attenuates mumps virus neurovirulence. Microbes and infection. 2015;17(3):228–36. doi: 10.1016/j.micinf.2014.11.006 . 25479555

[48] Bernardin F, Stramer SL, Rehermann B, Page-Shafer K, Cooper S, Bangsberg DR, et al. High levels of subgenomic HCV plasma RNA in immunosilent infections. Virology. 2007;365(2):446–56. doi: 10.1016/j.virol.2007.04.003 ; PubMed Central PMCID: PMC2001282. 17493654 PMC2001282

[49] Cheroni C, Donnici L, Aghemo A, Balistreri F, Bianco A, Zanoni V, et al. Hepatitis C Virus Deletion Mutants Are Found in Individuals Chronically Infected with Genotype 1 Hepatitis C Virus in Association with Age, High Viral Load and Liver Inflammatory Activity. PLoS One. 2015;10(9):e0138546. doi: 10.1371/journal.pone.0138546 ; PubMed Central PMCID: PMC4583497. 26405760 PMC4583497

[50] Karamichali E, Chihab H, Kakkanas A, Marchio A, Karamitros T, Pogka V, et al. HCV Defective Genomes Promote Persistent Infection by Modulating the Viral Life Cycle. Front Microbiol. 2018;9:2942. doi: 10.3389/fmicb.2018.02942 ; PubMed Central PMCID: PMC6287115. 30559733 PMC6287115

[51] Noppornpanth S, Smits SL, Lien TX, Poovorawan Y, Osterhaus AD, Haagmans BL. Characterization of hepatitis C virus deletion mutants circulating in chronically infected patients. J Virol. 2007;81(22):12496–503. doi: 10.1128/JVI.01059-07 ; PubMed Central PMCID: PMC2168980. 17728237 PMC2168980

[52] Pacini L, Graziani R, Bartholomew L, De Francesco R, Paonessa G. Naturally occurring hepatitis C virus subgenomic deletion mutants replicate efficiently in Huh-7 cells and are trans-packaged in vitro to generate infectious defective particles. J Virol. 2009;83(18):9079–93. doi: 10.1128/JVI.00308-09 ; PubMed Central PMCID: PMC2738267. 19587042 PMC2738267

[53] Lopez-Jimenez AJ, Clemente-Casares P, Sabariegos R, Llanos-Valero M, Bellon-Echeverria I, Encinar JA, et al. Hepatitis C virus polymerase-polymerase contact interface: significance for virus replication and antiviral design. Antiviral Res. 2014;108:14–24. doi: 10.1016/j.antiviral.2014.04.009 . 24815023

[54] Marukian S, Jones CT, Andrus L, Evans MJ, Ritola KD, Charles ED, et al. Cell culture-produced hepatitis C virus does not infect peripheral blood mononuclear cells. Hepatology. 2008;48(6):1843–50. doi: 10.1002/hep.22550 ; PubMed Central PMCID: PMC2592497. 19003912 PMC2592497

[55] Agudo R, Ferrer-Orta C, Arias A, de la Higuera I, Perales C, Perez-Luque R, et al. A multi-step process of viral adaptation to a mutagenic nucleoside analogue by modulation of transition types leads to extinction-escape. PLoS Pathog. 2010;6(8):e1001072. doi: 10.1371/journal.ppat.1001072 ; PubMed Central PMCID: PMC2928812. 20865120 PMC2928812

[56] Sanchez G, Bosch A, Gomez-Mariano G, Domingo E, Pinto RM. Evidence for quasispecies distributions in the human hepatitis A virus genome. Virology. 2003;315(1):34–42. doi: 10.1016/s0042-6822(03)00483-5 . 14592757

[57] Clemente-Casares P, Lopez-Jimenez AJ, Bellon-Echeverria I, Encinar JA, Martinez-Alfaro E, Perez-Flores R, et al. De novo polymerase activity and oligomerization of hepatitis C virus RNA-dependent RNA-polymerases from genotypes 1 to 5. PLoS One. 2011;6(4):e18515. doi: 10.1371/journal.pone.0018515 ; PubMed Central PMCID: PMC3072391. 21490973 PMC3072391

[58] Vo NV, Tuler JR, Lai MM. Enzymatic characterization of the full-length and C-terminally truncated hepatitis C virus RNA polymerases: function of the last 21 amino acids of the C terminus in template binding and RNA synthesis. Biochemistry. 2004;43(32):10579–91. doi: 10.1021/bi049773g . 15301555

