# Supplementary figures and images for: Guanosine inhibits hepatitis C virus replication and increases indel frequencies, associated with altered intracellular nucleotide pools
Source: PLoS Pathog. 2022 Jan 27;18(1):e1010210. doi: 10.1371/journal.ppat.1010210 (PMC8794218; doi:10.1371/journal.ppat.1010210)

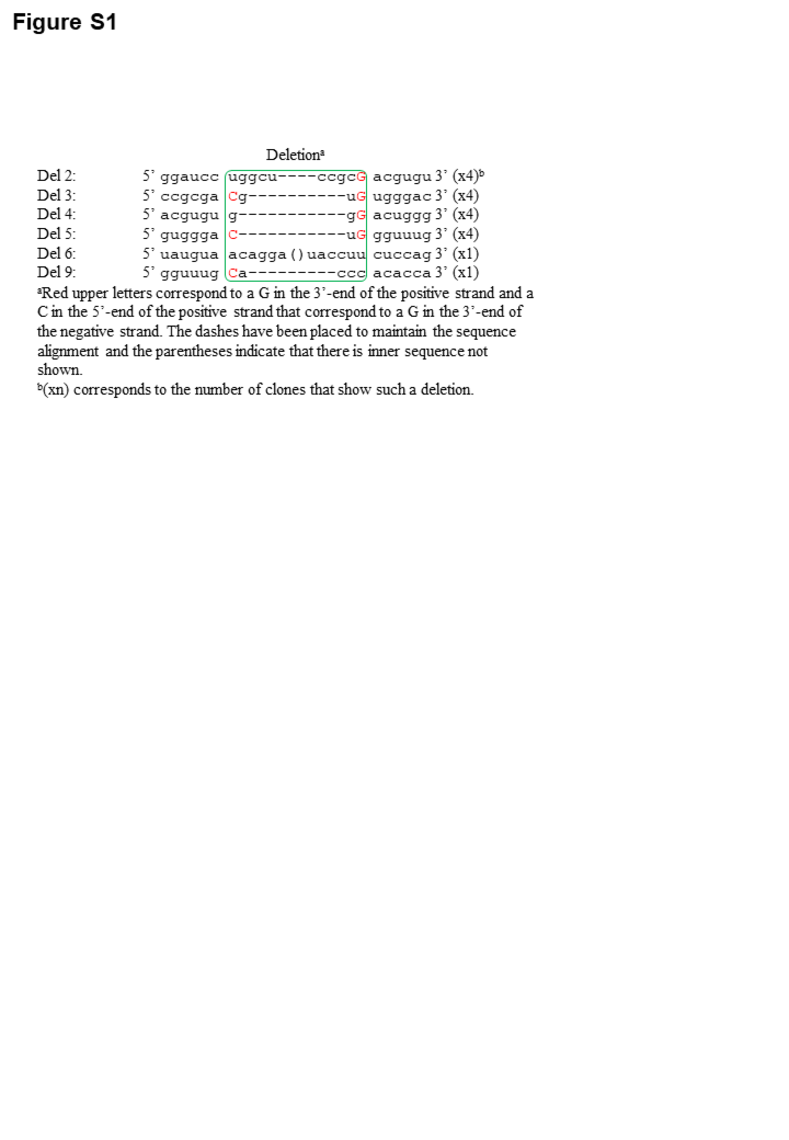

Supplement: S1 Fig — (TIF) [file ppat.1010210.s001.tif]

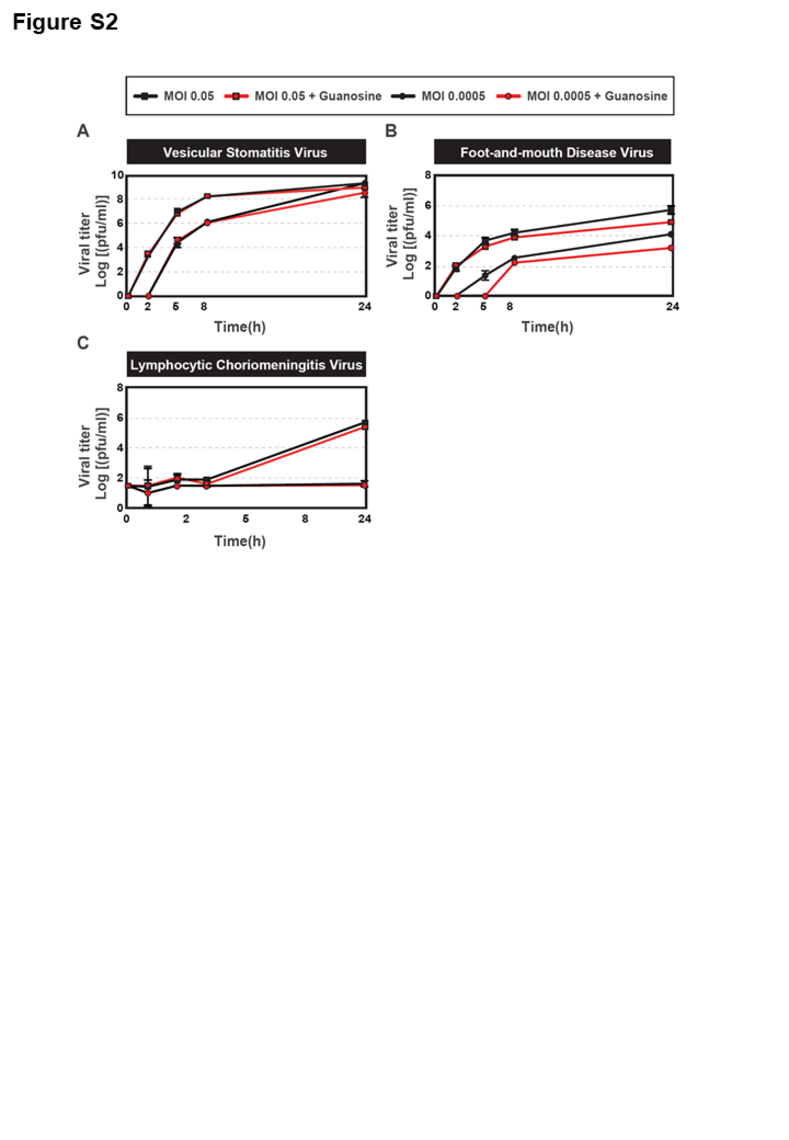

Supplement: S2 Fig — BHK21c2 cells were seeded and 16 hours prior to infection cells were untreated or treated with 800 μM of guanosine. Cells were infected with two different m.o.i. (0.05 or 0.0005), supernatants were collected at different times after infection (0-, 2-, 5-, 8- and 24-hours post-infection) and viral progeny production was analyzed. The m.o.i. and the treatment used are indicated in the upper box. The viral titer expressed as log10 pfu/ml is indicated in the ordinate. The time post-infection is indicated in the abscissa expressed in hours. (A) Effect of the addition of 800 μM of guanosine on VSV infectious progeny production. The differences between the slope of the treated and untreated has been calculated using an ANCOVA test in GraphPad (m.o.i. 0.05 p-value = 0.86; m.o.i. 0.0005 p-value = 0.62). (B, C) Same as A but the virus used are FMDV (B) and LCMV (C). Statistical differences between slopes were not found for FMDV: (m.o.i. 0.05 p-value = 0.45; m.o.i. 0.0005 p-value = 0.26) and for LCMV: (m.o.i. 0.05 p-value = 0.58; m.o.i. 0.0005 p-value = 0.79). Titrations were carried out in triplicate. (TIF) [file ppat.1010210.s002.tif]
